# Occipital Intralobar fasciculi: a description, through tractography, of three forgotten tracts

**DOI:** 10.1038/s42003-021-01935-3

**Published:** 2021-03-30

**Authors:** Maeva Bugain, Yana Dimech, Natalia Torzhenskaya, Michel Thiebaut de Schotten, Svenja Caspers, Richard Muscat, Claude J. Bajada

**Affiliations:** 1 Department of Physiology and Biochemistry, Faculty of Medicine and Surgery, The University of Malta, Msida, Malta; 2 Department of Cognitive Sciences, Faculty of Media and Knowledge Sciences, The University of Malta, Msida, Malta; 3 Brain Connectivity and Behaviour Laboratory, Sorbonne Universities, Paris, France; 4 Groupe d’Imagerie Neurofonctionnelle, Institut des Maladies Neurodégénératives -UMR 5293, CNRS, CEA University of Bordeaux, Bordeaux, France; 5 Institute of Neuroscience and Medicine (INM-1), Research Centre Juelich, Juelich, Germany; 6 Institute for Anatomy I, Medical Faculty, Heinrich-Heine-University Duesseldorf, Duesseldorf, Germany

**Keywords:** Neuroscience, Visual system

## Abstract

Diffusion MRI paired with tractography has facilitated a non-invasive exploration of many association, projection, and commissural fiber tracts. However, there is still a scarcity of research studies related to intralobar association fibers. The Dejerines’ (two of the most notable neurologists of 19^th^ century France) gave an in-depth description of the intralobar fibers of the occipital lobe. Unfortunately, their exquisite work has since been sparsely cited in the modern literature. This work gives a modern description of many of the occipital intralobar lobe fibers described by the Dejerines. We perform a virtual dissection and reconstruct the tracts using diffusion MRI tractography. The dissection is guided by the Dejerines’ treatise, *Anatomie des Centres Nerveux*. As an accompaniment to this article, we provided a French-to-English translation of the treatise portion concerning five intra-occipital tracts, namely: the stratum calcarinum, the stratum proprium cunei, the vertical occipital fasciculus of Wernicke, the transverse fasciculus of the cuneus and the transverse fasciculus of the lingual lobule of Vialet. It was possible to reconstruct all but one of these tracts. For completeness, the recently described sledge runner fasciculus, although not one of the Dejerines’ tracts, was identified and successfully reconstructed.

## Abstract (web summary)

Bugain et al perform a virtual dissection and reconstruct the occipital intralobar tracts using diffusion MRI tractography. They provide a modern anatomical depiction of what had originally been described by the Dejerines in the 19^th^ century, as well as a complete translation of the original text.

## Introduction

The advent of in vivo magnetic resonance imaging (MRI) techniques has heightened the desire to construct a comprehensive atlas of the human brain connective network—the so-called human connectome^1–3^. In humans, diffusion MRI paired with tractography permits a noninvasive three-dimensional reconstruction of large-scale white matter system organisation, which can then be compiled into an atlas^4^ (see reviews: refs. ^5–7^). Diffusion MRI has contributed to the verification of many association fiber pathways^8,9^, as well as to the redefinition of their boundaries and relations^10–17^. Recent success in discovering novel or forgotten association fibers has stoked interest in the less conspicuous short-range fiber connections, which can be more challenging to delineate^18–21^.

Despite the rapid advancement in the technology and the connectome mapping techniques available, there is a paucity in research related to short-range association fibers in the occipital lobe. The initial focus of this new field was geared towards the more prominent, and seemingly more physiologically crucial, long-range association fibers. A more generalised issue with MRI-based tractography studies is that the findings tend to remain speculative, devoid of tangible experimental evidence and ground truth, unlike those of axonal tracer studies^22^.

To partially overcome these inherent limitations, contemporary researchers can guide their findings through postmortem dissection studies, by consulting the tract tracing literature or historical neuroanatomy texts. The 19th-century neurologists were masterfully skilled in gross dissection and fastidious in the documentation of their composite observations. Numerous diffusion tractography studies have successfully used these classical dissections to interpret imaging results and identify false positives or false negatives^18,23–28^. Pioneering texts have also often been an initial source of inspiration for explorative neuroanatomical studies^18,23^. Indeed, we had previously studied dissections by 19th-century neurologists Joseph and Augusta Dejerine with a view to enriching the field’s knowledge and perspectives on long-range association fiber anatomy^29^. It showcased the pertinence of these historical studies and prompted further examination into the Dejerines’ other work on association short-range occipital fibers.

One of the biggest divisions across the neuroanatomical field towards the end of the 19th century was the definition and categorisation of association fibers. Prominent authorities at the time, such as Meynert, postulated that true association fibres only projected in an anterior-posterior dierection^18,30^, while Wernicke defended the existence of dorsal-ventral projecting association fibres, particularly in the occipital region^18,31,32^. Other neurologists, like the Dejerines and Sachs, supported the school of thought of Wernicke by acknowledging the existence of the vertical occipital fasciculus of Wernicke (VOF)^33,34^ and several other intralobar tracts with a dorsal-ventral trajectory. Whereas the work of Heinrich Sachs on the anatomy of intralobar pathways has previously been explored and translated^25,31^, the equally insightful contributions and perspectives of the Dejerines have yet to be explored and made more accessible.

Joseph Jules Dejerine (1849–1917) and his wife, Augusta Dejerine-Klumpke (1859–1927) frequently collaborated and are both individually celebrated as two of France’s most renowned 19th-century neurologists^29,35,36^. Arguably, the Dejerines’ seminal work is their two-volume neuroanatomical treatise, *Anatomie des Centres Nerveux*, originally published in 1895 and 1902 and then reprinted in 1980 by Mason-Elsevier^33,37^. The Dejerines’ impressive histological sections, accompanied by beautifully intricate illustrations by H. Gillet, are relevant even in this technological era, and are reference points for anatomical findings^26,38^. Of particular interest to us, the Dejerines dedicated a portion of the first volume to occipital intralobar association fibers. The Dejerines observed five fiber bundles specific to the occipital lobe: the stratum calcarinum, the VOF, the transverse bundle of the lingual lobule of Vialet, the transverse fasciculus of the cuneus and the stratum proprium cunei. Over 100 years later and these short, intralobar, association tracts remain poorly understood and surrounded by controversies due to inconsistent classification and nomenclature^18,39^.

Of late, the VOF, the largest of the occipital intralobar fibers dissected by the Dejerines, has been the focus of an increasing number of papers^10,16,18,40–42^. As of December 2019, a PubMed search generated thirty hits relating to the “vertical occipital fasciculus” when used as a key term. In comparison, the other intraoccipital tracts generate no results and remain overlooked, mostly being listed in passing (Table 1). An article by our group revisited the Dejerines’ work and provided access to an English translation of the portion of the Dejerines’ book related to the long-range association fibers of the human brain^29^. Here, we provide the complete translation of the description by the Dejerines of the occipital originating short-range fibers trajectories, from the original French taken from *Anatomie des Centres Nerveux* (1895 p. 780–784) (Supplementary note). The aim of this study is to use the observations made by the Dejerines for guidance through a virtual dissection of the intralobar tracts of the occipital lobe, based on in vivo diffusion imaging data. The virtual dissections enable us, to better discern the three-dimensional relations of these once-forgotten tracts.

**Table 1:** Summary of studies^10,12,16–18,24,25,31,32,34,41,42,44–46,51–53,56,59,62,63,91–99^ commenting on the intralobar occipital fibers in human or Old-World monkeys.

Intralobar occipital fibers	Studies identifying the fiber pathways	Studies mentioning the fiber pathways
Stratum calcarinum	Déjerine & Déjerine (1892), Koutsarnakis et al. (2019), Sachs & Wernicke (1892), Schmahmann & Pandya (2006), Takemura et al. (2020), Vergani et al. (2014), Vialet (1893), Von Boin et al. (1942)	Barker (1899), Campbell et al. (1905), Forkel et al. (2015), Maunsell & Van Essen (1983), Vergani et al. (2014)
Stratum proprium cunei	Déjerine & Déjerine (1892), Sachs & Wernicke (1892), Schmahmann & Pandya (2006), Vergani et al. (2014), Vialet (1893)	Barker (1899), Campbell et al. (1905), Forkel et al. (2015), Greenblatt (1973), Vergani et al. (2014)
Sledge runner fasciculus	Güngör et al. (2017), Koutsarnakis et al. (2019), Muftah Lahirish et al. (2020), Vergani et al. (2014)	
Transverse fasciculus of the cuneus	Dejerine & Dejerine (1892), Sachs & Wernicke (1892), Schmahmann & Pandya (2006), Vergani et al. (2014), Vialet (1893), Von Boin et al. (1942)	Barker (1899), Campbell et al. (1905) Forkel et al. (2015), Greenblatt (1973), Takemura et al. (2019), Vergani et al. (2014), Yeatman et al. (2014)
Transverse fasciculus of the lingual lobule of vialet	Dejerine & Dejerine (1892), Schmahmann & Pandya (2006), Vialet (1893), Von Boin et al. (1942)	Barker (1899), Campbell et al. (1905), Greenblatt (1973), Takemura et al. (2019), Yeatman et al. (2014)
Vertical occipital fasciculus	Bartsch et al. (2013), Briggs et al. (2018), Dejerine & Dejerine (1892), Duan et al. (2015), Güngör et al. (2017), Keser et al. (2016), Martino & García-Porrero (2013), Muftah Lahirish et al. (2020), Oishi et al. (2018), Palejwala et al. (2019), Panesar et al. (2019), Rokem et al. (2017), Sachs & Wernicke (1892), Schmahmann & Pandya (2006), Schurr & Mezer (2019), Takemura et al. (2016), Takemura et al. (2017), Takemura et al. (2020), Vergani et al. (2014), Vialet (1893), Von Boin et al. (1942), Weiner et al. (2017), Wernicke (1876), Wu et al. (2016), Yeatman et al. (2013), Yeatman et al. (2014)	Barker (1899), Campbell et al. (1905), Forkel et al. (2015), Greenblatt (1973), Vergani et al. (2014)

## Results

**Fig. 1:**
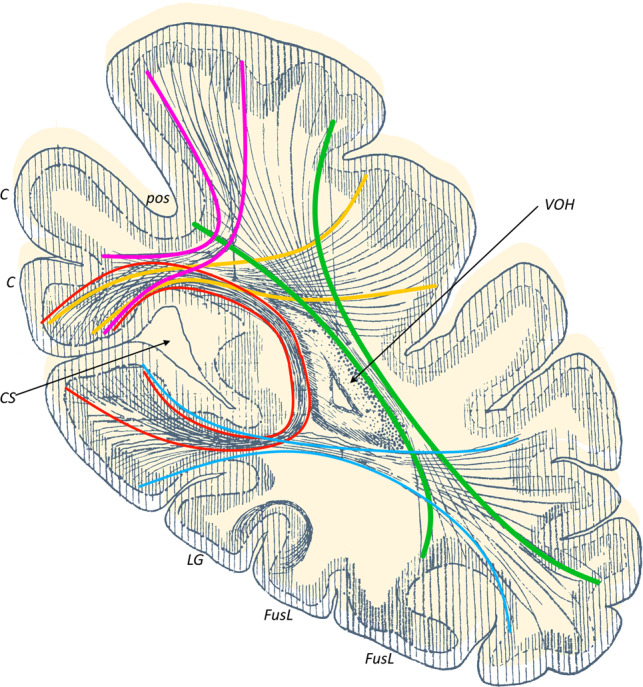
The Dejerines’ intraoccipital fibres. A coronal slice of the occipital lobe depicting the five occipital intralobar fibres identified by Dejerine. Green, vertical occipital fasciculus; blue, transverse fasciculus of the lingual lobule of Vialet; red, stratum calcarinum; magenta, stratum proprium cunei; yellow, transverse fasciculus of the cunei; C cuneus, CS calcarine sulcus, LG lingual gyrus, FusL fusiform lobule, pos parieto-occipital sulcus, VOH occipital horn of the lateral ventricle. Image adapted and reproduced from the illustration by H. Gillet in Dejerine & Dejerine (1895, p. 781).

**Fig. 2:**
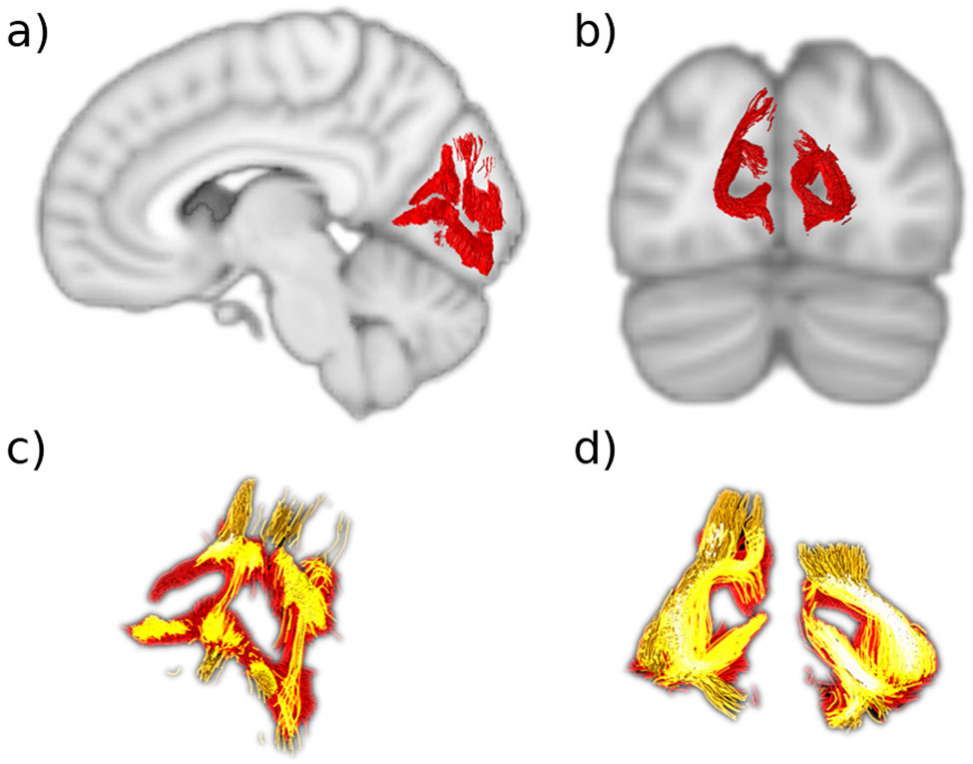
The stratum calcarinum (SC). **a** Sagittal slice showing the right SC (red) curved around the calcarine fissure. **b** Coronal slice showing the left and right SC. **c**, **d** The two layers of the SC. The deeper and longer layer (orange) lies under the more superficial and u-shaped layer (red). Where they intersect is shown in bright yellow.

**Fig. 3:**
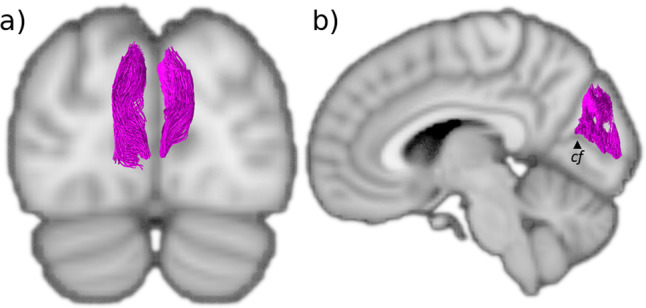
The stratum proprium cunei (SPC). **a** Coronal view of the SPC (magenta) that lines the midline of the cuneus. **b** Sagittal view of the SPC showing how it arises from the superior bank of the calcarine fissure. cf: calcarine fissure.

**Fig. 4:**
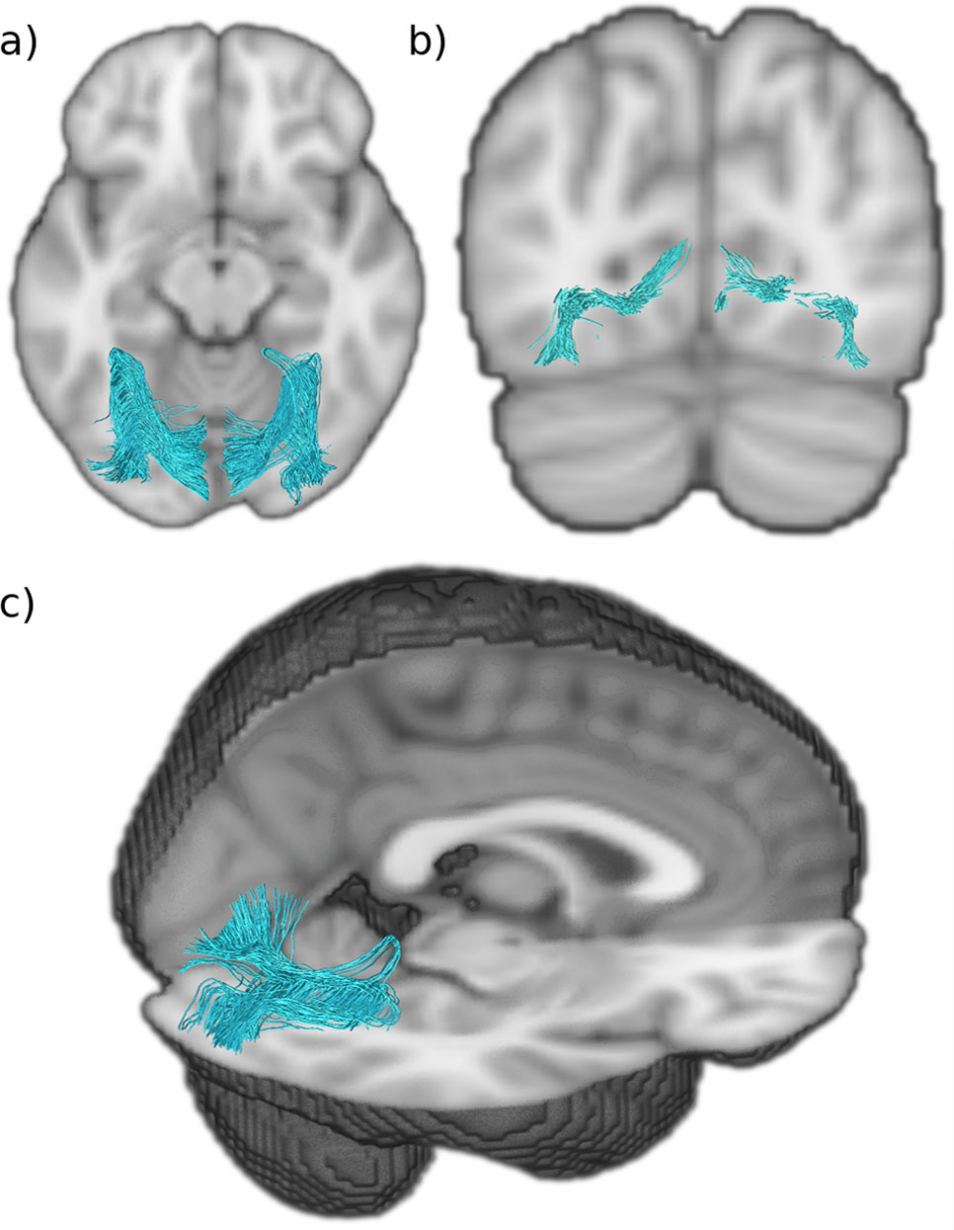
Putative transverse fasciculus of the lingual lobule of Vialet (TFV). **a** Axial view of the TFV (blue) showing it projects anteriorly before it reflects in the anterior aspect of the lingual gyrus. **b** Coronal view of TFV showing it arises from the inferior gyri of the calcarine fissure and terminates infero-laterally. **c** Sagittal and axial view of the TFV showing how it does a hairpin loop at the anterior edge of the lingual gyrus.

**Fig. 5:**
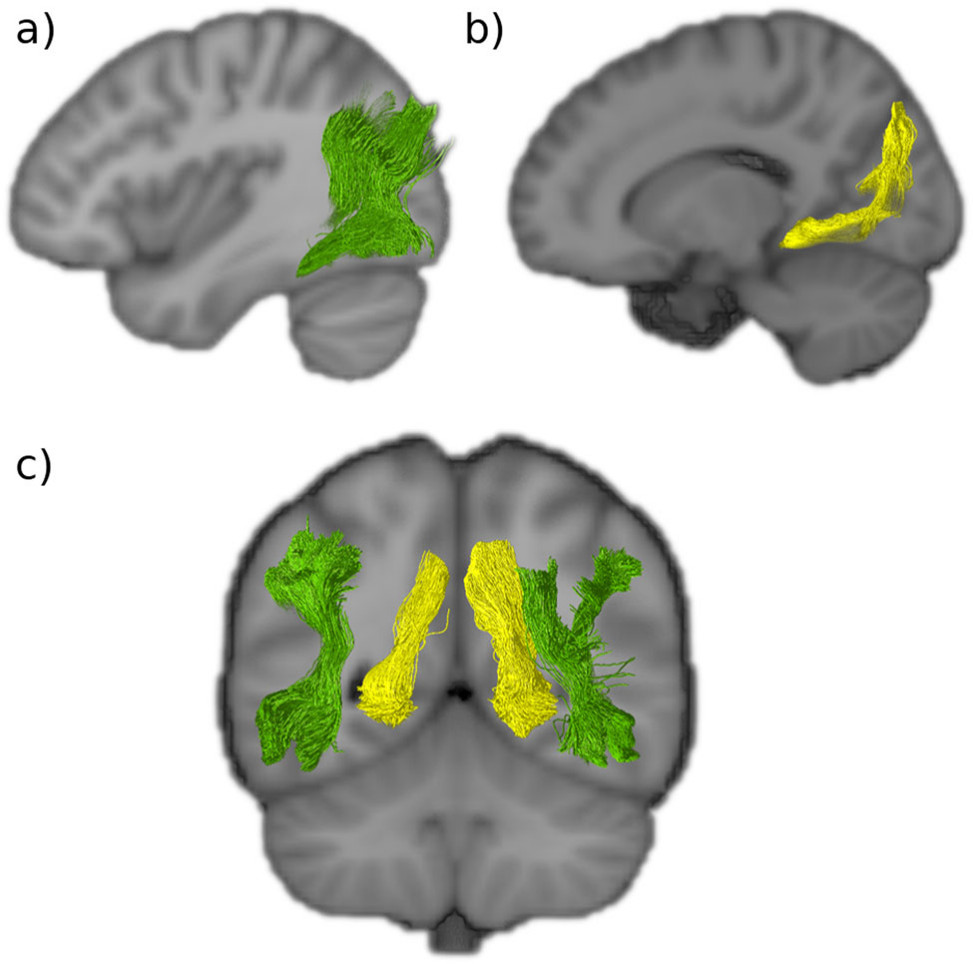
Dorsal to ventral intralobar fibers. **a** Sagittal view of the right vertical occipital fasciculus (VOF in green). **b** Sagittal view of the right sledge runner fasciculus (SRF in yellow). **c** Coronal slice of the VOF and SRF showing how they lie lateral and medial to the lateral ventricles, respectively. Both connect dorsal aspects of the occipital lobe to ventral aspects. VOF vertical occipital fasciculus, SRF sledge runner fasciculus.

**Fig. 6:**
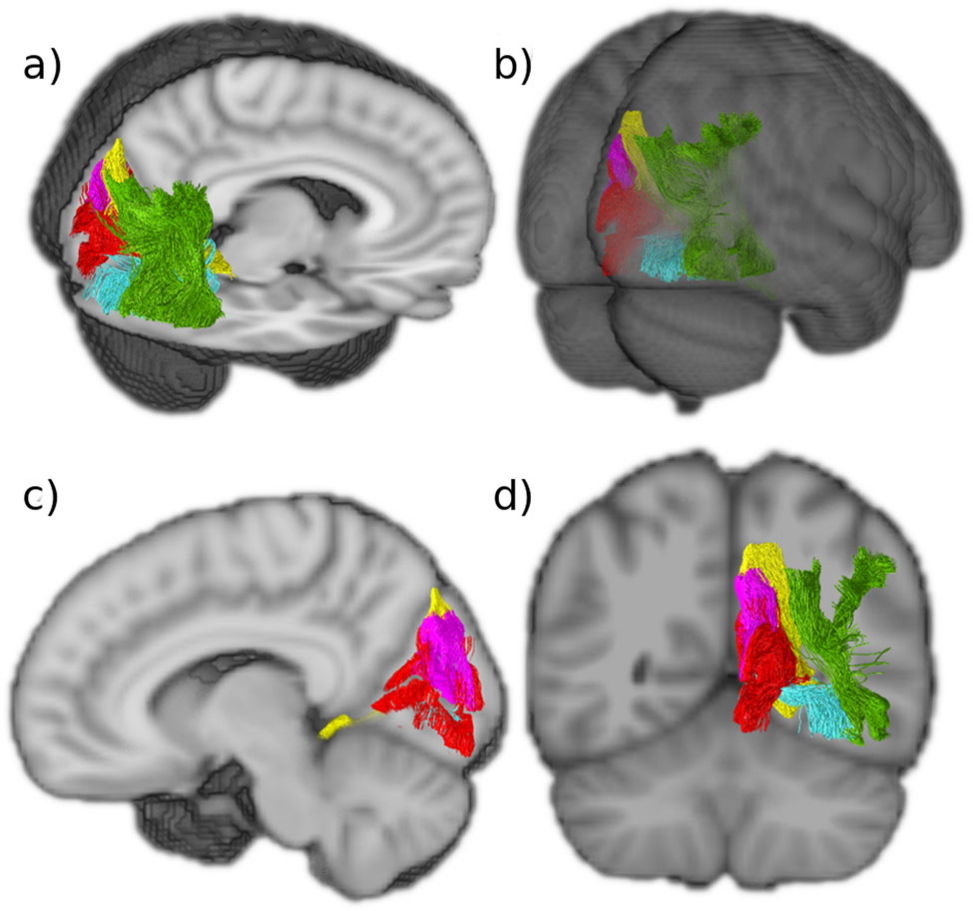
The five intralobar fibers identified in the occipital lobe. Tracts generated from group averaged data of 24 subjects. **a** Sagittal and axial view. **b** Posterior view. **c** Sagittal view showing that the SPC is more medial than the SRF and that the SC extends more widely than the other medial intralobar tracts. **d** Coronal view showing that the intralobar fibers connect all aspects of the occipital lobe. VOF vertical occipital fasciculus (green), SRF sledge runner fasciculus (yellow), SPC stratum proprium cunei (magenta), SC stratum calcarinum (red), TFV putative transverse fasciculus of the lingual lobule of Vialet (blue).

**Fig. 7:**
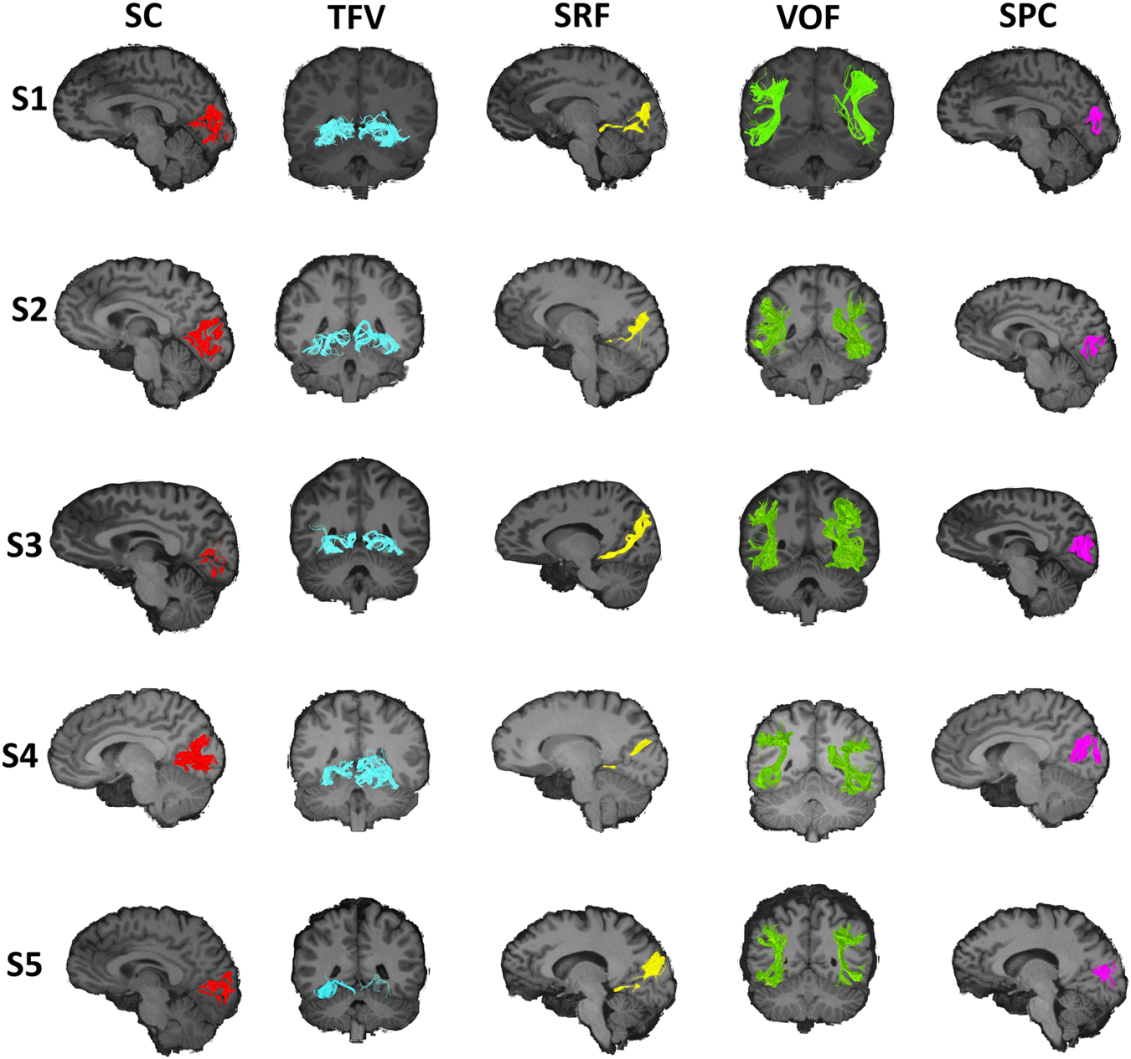
Intralobar fibers in the occipital lobe presented in individual subjects. Representation of the identified occipital intralobar tracts in five of the 24 subjects used in this study. The same methodology and regions of interest (ROIs) used to isolate the tracts in the group averaged tractography data. The ROIs were wrapped to native subject spaces. This generates tractography findings that support the presence of the tracts in distinct individuals and depict the subject specific trajectory of the intralobar tracts. VOF vertical occipital fasciculus (green), SRF sledge runner fasciculus (yellow), SPC stratum proprium cunei (magenta), SC stratum calcarinum (red), TFV putative transverse fasciculus of the lingual lobule of Vialet (blue).

Using constrained spherical deconvolution (CSD) tractography, this study successfully identified and mapped four of the five occipital intralobar white matter tracts observed by the Dejerines (Fig. 1). The translation of the Dejerines’ work on these fibers, from 19th-century French to accessible English (Supplementary Note), helped guide the tractography findings. Tractography reconstruction of the occipital intralobar tracts have been overlaid on a 3D T1-weighted MNI template image and presented in Figs. 2–5. The relation of each tract to each other are provided in Fig. 6, while Fig. 7 shows individual tracts in a representative sample of the subjects.

The following descriptions of the tracts are based on a virtual dissection derived from averaged orientation distribution functions (CSD ODFs) from 24 healthy subjects (Figs. 2–6). To probe the individual level results, the same dissections were also performed on the individual level data. The individualized dissections were found to be generally consistent with the average dissection (see Fig. 7 for a representative sample or the online material for the full dataset).

### Occipital diffusion tractography

With regard to short-range association fibers arising from the occipital lobe, the Dejerines described the vertical occipital fasciculus (VOF), which spans the entirety of the occipital lobe lateral to the posterior ventricle, and four shorter association fiber tracts projecting superiorly and inferiorly from the banks of the calcarine fissure: the stratum calcarinum (SC), stratum proprium cunei (SPC), transverse fasciculus of the cuneus (TFC), and the transverse fasciculus of the lingual lobule of Vialet (TFV) (Fig. 1).

### Stratum calcarinum (SC)

The stratum calcarinum (SC) consists of several U-shaped fibers that connect the two banks of the calcarine fissure, a major landmark that medially divides each occipital lobe into the wedge-shaped cuneus superiorly, and the lingual lobule inferiorly, and represents the boundaries of the primary visual cortex. This group of U-shaped fibers extends all along the calcarine fissure from the occipital pole to its junction with the parieto-occipital fissure at anterior border of the occipital lobe (Fig. 2). The SC is an unusually large U-fiber system because it directly connects adjacent gyri across a long and deep fissure—perhaps the reason the Dejerines devoted considerable attention to it, describing it alongside other intralobar fibers.

The SC fibers are composed of two superimposed layers (Fig. 2c). The first, most superficial layer has a vertical and horizontal orientation that embraces the superior and inferior edges of the fissure. The second has longer and more deeply penetrating fibers that join the medial-central aspects of the cuneus to the medial-central aspects of the lingual gyrus. SC layers lie directly on top of the longitudinal fibers of the inferior longitudinal fasciculus that joins them to carpet the floor of the calcarine fissure. These fibers run perpendicular to the vertical SC fibers and interlink the different U-fiber units along the fissure like rails on a track. This relation between the inferior longitudinal fasciculus and the U-fibers of the occipital lobe was depicted by Ffytch and Catani^43^, however, they did not explicitly mention the SC system or distinguish it from other U-fibers.

The horizontal aspect of the SC projects away from the midline into the bank of the fissure and becomes intertwined with the other intralobar tracts that originate from the lips of the calcarine fissure: the stratum proprium cunei in the superior lip and the putative transverse fasciculus of the lingual lobule of Vialet in the inferior lip. In the anterior part of the fissure, the vertical portion of the SC is on the same plane, and slightly overlaps the sledge runner fibers (not described by the Dejerines) that travel vertically downwards, beneath the calcarine fissure, prior to continuing transversely to the anterior lingual gyrus (Fig. 6).

### Stratum proprium cunei (SPC)

The stratum proprium cunei (SPC) arises from the superior bank of the anterior calcarine fissure, just posterior to the junction with the parieto-occipital sulcus (Fig. 3). The tract connects the inferior aspect of the cuneus with the most anteromedial convexity of the occipital lobe, just posterior to the precuneus. The SPC runs deep to the parieto-occipital sulcus, at the level of the calcar avis, initially coursing laterally away from the calcarine fissure for a short distance, before ascending vertically to terminate in what is classically referred to as the first occipital convolution. Unlike the vertical occipital fasciculus that fans out across the entirety of the first occipital convolution, the SPC projections remain on a plane medial to the posterior horn of the ventricle. Although the SPC connects distal regions of the same lobe, it is a relatively short and narrow association fiber tract. Our findings align with the Dejerines’ and show that the SPC runs neatly along the anteromedial margins but is constrained to the occipital lobe.

### Transverse fasciculus of the cuneus of Sachs (TFC)

The transverse fasciculus of the cuneus of Sachs (TFC) is described as a tight, slender bundle, consisting of relatively few fibers that initially move away from the upper fold of the calcarine fissure with a strong oblique trajectory. They course laterally and superiorly to the roof of the posterior lateral ventricle and cross the cuneus to terminate in the lateral convexities of the occipital lobe^24,25,34,44–46^. Unfortunately, in the present study, it was not possible to identify a bundle resembling the TFC.

### Transverse fasciculus of the lingual lobule of Vialet (TFV)

A tract resembling the many aspects of the Dejerines’ description of the transverse fasciculus of the lingual lobule of Vialet (TFV) was successfully isolated (Fig. 4). The Dejerines depicted the TFV as a mirror counterpart to the transverse fasciculus of the cuneus, as it connects the inferior gyrus of the calcarine fissure to the infero-lateral aspects of the occipital lobe, akin to the way in which the transverse fasciculus of the cuneus connects the fissure to the external surface of the cuneus. In contrast, the stratum proprium cunei connects the fissure to the medial surface of the hemisphere and does not appear to have an inferior lingual gyrus equivalent. Interestingly, the putative TFV origin can be found all along the calcarine fissure with a span similar to the stratum proprium cunei.

The putative TFV has a morphology that resembles an extended U-fiber; the tract travels laterally away from the fissure prior to adopting an intense anterior-oblique trajectory that sharply curves back on itself and continues in a posterior-oblique course toward the lateral convexity of the occipital lobe. Due to the squatness of the lingual lobule, the tract appears quite flat and two ends of the fiber tract are nearly on the same coronal plane, with the lateral terminations slightly inferior to its calcarine fissure origin. The putative TFV starts superiorly and medially to the inferior longitudinal fasciculus and occipital projections of the inferior fronto-occipital fasciculus, and crosses both laterally and superior to these two major association fiber bundles, before descending just laterally to them.

### Vertical occipital fasciculus (VOF)

Using the Dejerines’ classical description, we identified the vertical occipital fasciculus (VOF) (Fig. 5a), and it aligns well with modern tractography findings. Of the five occipital tracts the Dejerines observed, the VOF is the longest and most prominent, and consequently has been the focus of many more studies. Often described as a short, vertical, and ‘sheet-like’ bundle of fibers, the VOF “connects the superior aspect of occipital lobe to its inferior surface” (Dejerine & Dejerine-Klumpke^33^, p. 779) maintaining a dorsal-ventral course lateral to the posterior ventricle. More specifically, the inferior terminations of the VOF extend from the ventral temporo-occipital cortex region, encompassing the inferior temporal gyrus and its intersection with the anterior aspects of the middle and inferior occipital gyri. The superior projections of the VOF reach the lateral gyri of the occipital lobe, including the transverse occipital sulcus, with several fibers reaching the posterior end of the intraparietal sulcus and the angular gyrus.

The ‘sheet-like’ stem of the VOF tract, that courses through the central part of the occipital lobe, is a wide bundle that has a predominantly vertical trajectory, apart from the ventral and dorsal terminations that flick out laterally and transversely to reach the convexity of the lateral occipital lobe across a wide area. Whereas the stratum calcarinum is covered laterally by the inferior longitudinal fasciculus, the VOF passes laterally to the inferior longitudinal fasciculus along with the inferior fronto-occipital fasciculus, continuing superiorly to both and coursing vertically, posterior to the arcuate fasciculus.

### Sledge runner fasciculus (SRF)

Tracts resembling the recently discovered sledge runner fasciculus (SRF)^25^ were identified in both hemispheres and reconstructed successfully (Fig. 5b, c). The SRF connects the posterior precuneus to the anterior lingual gyrus of the lingual lobule, taking a strongly inferior anterior-oblique trajectory with a couple of convexities—giving it a sledge-like morphology. It is a long occipital tract that traverses from the dorsal to ventral aspects of the cerebrum like the vertical occipital fasciculus. However, unlike the vertical occipital fasciculus, it is medial to the ventricles. With a compact central stalk that widens as the tract descends anteriorly and narrows superiorly, it is a prominent bundle of similar length to the vertical occipital fasciculus. As in the first observations of the SRF obtained through fiber microdissections, the results showed that the dorsal SRF terminations were located at the anterior border of the cuneus, adjacent to the midline of the cerebrum. The dorsal SRF terminations are entangled with the anterior fibers of the stratum proprium cunei bundle (Fig. 6). The ventral terminations are more anteriorly located and project deep into the lingual gyrus, posterior to the cingulate isthmus and the posterior part of the parahippocampal gyrus.

## Discussion

The anatomical findings described in the present study suggests that the occipital lobe has at least five intrinsic white matter structures that fall between the long association fibers and the very short U-shaped fibers. These are: the stratum calcarinum, the transverse fasciculus of the lingual lobule of Vialet, the stratum proprium cunei, the vertical occipital fasciculus of Wernicke (VOF)—which were described by the Dejerines, and the recently discovered sledge runner fasciculus (SRF). Apart from the vertical occipital fasciculus of Wernicke and the sledge runner fasciculus, none have been the focus of dedicated studies since the turn of the 20th century. Certain, expansive, U-fibers studies have previously presented findings that are suggestive of some of the shorter occipital intralobar fibres identified in this current study^47–50^. Yet, the scope of these U-fiber studies is vast and they do not provide precise descriptions of the trajectory of the occipital U-fiber tracts. This makes it difficult to gauge to what extent pathways depicted in their figures are equivalent to our findings. As such, dedicated and focused anatomical studies are still required.

This current study also highlights the persistent utility of historical studies for guidance of neuroimaging findings.

### Vertical occipital fasciculus (VOF)

The VOF’s existence as an association fiber bundle has been extensively verified, through diffusion MRI (dMRI) studies^12,16,18,40,51^, postmortem dissections^25,41,52^, and it has even been identified and compared in macaques^53^. Yet, the boundaries of the pathway remain unclear and disputed^54^. Its vertical trajectory, like that of the stratum proprium cunei, distinguishes it from the other long association fibers that project into the occipital lobe, and its long dorso-ventral trajectory, located lateral to the ventricle, sets it apart from the intraoccipital tracts. The Dejerines, like other early neuroanatomists, admitted to poorly defining the anterior limits of the VOF. Their descriptions suggest that it extended anteriorly beyond the boundaries of the occipital lobe, descriptions borne out by most recent tractography studies, including this present one. Hence, it is arguable that the VOF is not strictly an autochthonous tract restricted to the occipital lobe^10^. Depending on how anterior the ventral terminations of the VOF were, it assumed either an oblique dorsal and posterior trajectory, or in the case of more posterior ventral terminations, a vertical trajectory to the occipital cortex. We did not find strong ventral VOF termination within the ventromedial-fusiform gyrus or the dorsomedial portion of the occipital lobe. Our findings show that the VOF extends from the dorsal to ventral aspects of the lateral parietal and occipital lobe as a thin sheet and terminates at the lateral-posterior end of the temporo-occipital region (Fig. 5). As described by the Dejerines, the VOF courses laterally to the occipital horn and the inferior longitudinal fasciculus.

Studies now aim to better differentiate the VOF fibers from the neighbouring larger bundles. The VOF projects superior to the inferior longitudinal fasciculus. Their proximity once made the VOF difficult to distinguish from the terminal portions of the inferior longitudinal fasciculus, leading to its misidentification in one of the early studies on the macaque^24^. Recent findings by Takemura et al.^53^ demonstrate a clear separation between the VOF and the inferior longitudinal fasciculus and optic radiations streamlines in humans through dMRI. This supports what had first been indicated in classical studies of the human and macaque by the Dejerines and Wernicke, respectively^33,55^.

Similarly, the anterior dorsal terminations of the VOF closely borders the posterior and the ventral aspect of the arcuate fasciculus^10,12,16,54^. This has generated debate about the anatomical boundaries and distinctive functions^17,56^ of VOF and arcuate fasciculus. The proximity of the VOF to the posterior arcuate fasciculus has been seen to be dependent on species, subject variability, local model, and tractography algorithms^10,57^.

Borders for the VOF, or any other occipital intralobar tract, can manifest small discrepancies across studies partly due to brain borders being a human construct, high subject variability, the limits of imaging resolution, and the larger number of more prominent tracts that cross the intralobar tracts^58^. The occipital intralobar tracts, notably the VOF, have a strong dorsal-ventral trajectory, whereas many of the major long association fiber pathways that enter the occipital lobe have an anterior-posterior trajectory. Hence, the intralobar fibers cross paths with more prominent bundles that can cancel out their signal. The Dejerines themselves remarked on the limits of even masterful brain dissection skills for identification of terminations in pathway-dense regions, limiting the extent of guidance that postmortem dissections can provide. The continuous improvement in MRI tractography angular resolution, and the development of alternative models, have made some headway^53,54,59^.

It is important to make these distinctions, for all association fibers, in order to generate a truly representative map—that is, one that does not include pathways that appear longer or wider than they truly are because they artificially contain subsections of adjacent tracts. Knowing the extent of a distinct white matter system is useful for future work concerning functional specialization and pathology. It has been suggested that certain U-fibers, situated between the VOF and other long association pathways, form artificial connections, or U-fiber ‘bridges’, between the pathways that can make the two tracts look like one larger tract^16^. The intermingling of U-fibers is known to obstruct the visualisation and interpretation of the deeper underlying subcortical tracts in both fiber tractography and dissections^60^.

The difficulty that arises when studying the work of the Dejerines, like other 19th-century anatomists, relates to translating their definition of structures into the current school of thought. Inconsistent landmarks, nomenclatures, and methods were used to define and visualise structures. This discordance makes it difficult to reference a classic text and persists to the present day. Nevertheless, the congruency between the Dejerines’ dissection and modern imaging suggests that the other tracts they described should also be successfully visualised in vivo.

### Stratum calcarinum (SC)

In vivo and in vitro dissections of the calcarine fissure reveal a large continuous bundle of U-fibers, named the stratum calcarinum (SC), connecting the upper and lower edges of calcarine cortex (Fig. 2). U-fibers typically do not form a clear pathway or large fiber system. The Dejerines chose to omit the SC from the portion of their book that concerns U-fibers, in favor of discussing it alongside the association fibers of the occipital lobe to highlight the prominence and size of the SC. The Dejerines emphasised that it is not a true association fiber or a single entity. Instead, it represents a series of tracts forming an oversized U-fiber system that curves around the whole calcarine fissure and is specific to the primary visual cortex. It extends from the posterior end of the occipital horn to the intersection with parieto-occipital fissure and circumnavigates the calcar avis in its full extension. The SC intermingles with the other association fibers like the transverse fasciculus of the lingual lobule of Vialet and stratum proprium cunei, which link the primary visual cortex with the convexity of the occipital lobe.

The SC system forms an atypical bundle as it consists of a long and deep layer underlying a short and more superficial layer. The superficial SC layer, as can be seen from the results (Fig. 2c), has short projections that tightly carpet the floor of the calcarine fissure and join the superior and inferior gyri that directly border the fissure. This gives the layer the characteristic ‘U’ appearance. The second underlying layer has wider projections and a weaker U shape. Its longer fibers connect the medial aspect of the central cuneus to the inferior-medial aspect of the lingual lobule.

The calcarine fissure receives several white matter bundle pathways that share similar orientations at the level of the fissure, making it particularly difficult to differentiate them. Campbell^46^, postulated that the majority of the fibers labelled by the Dejerines, such as the SC, were actually terminal fibers of the optic radiation. Indeed, the Dejerines^33^ acknowledged that the histological staining method employed was insufficient to confidently evaluate the degree to which association fibers, like the SC, contribute to the projection fiber pathways that are situated along the ventricles. However, more recent studies, conducted with the improved Klingler’s technique, are congruent with the Dejerines’ description of the SC^20,25^. Unfortunately, to date, most mentions of the human SC, transverse fasciculus of the lingual lobule of Vialet, transverse fasciculus of the cuneus, and stratum proprium cunei in the literature have been in reference to historical dissections (Table 1).

A human postmortem dissection study had shown the three-dimensional relationship between the sledge runner fasciculus and SC, with the sledge runner fasciculus running obliquely, almost perpendicularly, superior to the SC fibers that lie in the anterior portion of the calcarine fissure just posterior to the junction it shares with the parieto-occipital sulcus^20^. There is a similar overlap that occurs between the SC and the stratum proprium cunei (Fig. 6). The posterior inferior longitudinal fasciculus projects obliquely toward the calcarine fissure and passes superior to the SC, before descending and coating the internal wall of the occipital horn, separating the ventricle from the SC^24,33^.

The Dejerines considered the SC as one of the most important short-range connections in the occipital lobe. The proposed importance of the SC arises from its role in connecting the superior and inferior vision field regions all along the fissure, likely implicating it in the visual pathways of the macula and the retina. It has been postulated that the SC, stratum proprium cunei and transverse fasciculus of the cuneus contribute to coordination and propagation of the visual stimuli of color, shape, and motion from the primary cortex to higher order association areas, permitting both dorsal and ventral outflow to the rest of the parietal and temporal cortex^45^.

### Stratum proprium cunei (SPC)

The SPC is a short and seemingly inconsequential tract that rises in the sagittal plane from the calcarine fissure at the medial border of the hemisphere (Fig. 3). Since the first reports by Sachs^34^, it has only been vaguely mentioned in passing^24,25,45,46,61^. Our current findings align strongly with the Dejerines’ observation that the SPC “*originates from the superior lip of the calcarine fissure*” and projects dorsally to the “*cortical region of the superior border of the hemisphere*”^33^, (p. 782). The results show a bundle with a clear vertical projection that links the upper border of the anterior calcarine fissure to the medial aspect of the cuneus convexity. Like the stratum calcarinum, the SPC extends along the entire length of the calcarine fissure. It differs, however, by being limited to the cuneus. Schmahmann & Pandya^24^, speculated that the SPC bundle corresponds to fibers they identified in rhesus macaque, located in the superior parietal lobule and the dorsal occipital white matter, caudal to the dorsal component of the superior longitudinal fasciculus. The fibers connected the medial pre-occipital gyrus of the occipital lobe with the medial part of the superior parietal lobule. However, the SPC we observed does not extend past the parieto-occipital fissure and does not correspond with the observations of Schmahmann & Pandya^24^ who describe it as more anteriorly projecting compared to the system we have identified. The SPC shares a similar trajectory to the dorsal half of the stratum calcarinum and sledge runner fasciculus. This study specifically followed the Dejerines’ descriptions, which limits the SPC to the medial surface of the cuneus to differentiate the SPC from the stratum calcarinum and sledge runner fasciculus. In a postmortem dissection, Vergani et al.^25^ depict both the SPC and their discovery of the sledge runner fasciculus distinctively. Interestingly, another study examining the sledge runner fasciculus discusses its relation to the stratum calcarinum, but not the SPC. It is unclear as to whether the authors have interpreted the SPC as forming part of the sledge runner fasciculus^62^. Due to the limited literature on the SPC, it is not clear to what extent diffusion tractography’s limitations have affected the reconstruction.

### Transverse fasciculus of the lingual lobule of Vialet (TFV)

The Dejerines named the TFV after Nehemie Vialet who described it in detail and published a paper arguing for its existence as a distinct fascicle^44^. The Dejerines were able to successfully reproduce the observations made by Vialet in their own dissection studies. Our study succeeded in reconstructing in several individual subjects a putative TFV bundle (Fig. 7). This was unexpected, as like the transverse fasciculus of the cuneus, the TFV tract has not been mentioned in recent occipital fiber dissection or tractography studies. The TFV tract can be seen as a counterpart to the transverse fasciculus of the cuneus, as it connects the inferior gyri of the calcarine fissure to the convexity of the lingual lobule. The TFV can be differentiated from the inferior longitudinal fasciculus that courses alongside it at the level of the lingual gyrus and posterior temporal lobe, by its slightly thinner bulk and paler Pal-haematoxylin staining^33,44^. Vialet considered this bundle to be functionally important as it seems to represent the inferior portion of the occipital lobe association fiber system.

The existence of the TFV, like the transverse fasciculus of the cuneus and vertical occipital fasciculus, as a distinct entity was heavily contested shortly after its discovery. This resulted in their omission from much of the literature, such as the neuroatlases of notable and influential neurologists like that of Constantin Von Monakow. Von Monakow described various subcortical structures with the locations and gross trajectories that match the TFV, transverse fasciculus of the cuneus and vertical occipital fasciculus, but was unable to differentiate them from neighbouring bundles and so negated their existence as distinct tracts^18^. Indeed, Schmahman & Pandya^24^, professed uncertainty as to the existence of the TFV as a distinct tract and considered the TFV as described by the Dejerines to be equivalent to the *“transverse inferior longitudinal fasciculus fibers that lie within the ventral part of the occipital and temporal lobe and that link the lateral cortices with the medial cortices”* (p. 450). However, Schmahmann & Pandya^24^ had made a similar comment in relation to the vertical occipital fasciculus and had interpreted it as part of the vertical component of the inferior longitudinal fasciculus^23^, which was later disputed by more focused tractography studies. Vergani et al.^25^, conducted a thorough postmortem exploration of the intralobar fibers of the occipital lobe, in an effort to replicate the work of Sachs^34^. Interestingly, like Sachs, the authors did not mention a tract that matches the morphology of the TFV as described later by Vialet, the Dejerines, and as identified in this study. A recently resurfaced photograph of a Weigert-stained histological slide of the macaque occipital lobe by von Bonin et al.^61,63^ clearly depicts the vertical occipital fasciculus and stratum calcarinum, and somewhat less clearly, the TFV and transverse fasciculus of the cuneus named the fasciculus transversus lingualis and fasciculus transversus cunei, respectively^63^. The brevity of the description makes it hard to ascertain in greater detail the course of the TFV in the macaque.

Although identified in multiple subjects of this present study, limitations inherent to this study and past ones, puts into question the existence of the TFV and whether it is a misconstrued artifact formed in part by the optic radiations, or other such neighbouring pathways. Validation of the TFV will depend on future studies with higher resolution and varied methodology, and it remains putative until such a time.

### The transverse fasciculus of the cuneus (TFC)

Our study did not identify a consistent tract in either hemisphere that matches any previous descriptions. Yet, the Dejerines remarked that the calcarine cortex is attached to the cuneus and first occipital convolution through both the stratum proprium cunei and TFC and this observation of the TFC was also made by previous works^25,34,44–46,61^. The Dejerines, with reference to Sachs’ work, described the TFC as having a trajectory that “*runs slightly obliquely, anteriorly and laterally, and radiate[s] in the superior parietal lobule and in the angular gyrus*” (Dejerine & Dejerine-Klumpke^33^, pg. 781). It was specified by the Dejerines, Sachs and Vialet^33,34,44^ that the origin of the TFC is at the level of the calcarine fissure among the stratum calcarinum and stratum proprium cunei. This was more recently supported by Vergani et al., 2014,^25^ who conducted an in vitro dissection and identified segments of the TFC, which they labelled stratum transverse cunei. The cuneus is very compact with a high density of white matter fibers crossing it. Therefore, there are a large amount of fibers with an antero-posterior trajectory, crossing paths with the thinner TFC, either perpendicular or parallel to it, disrupting the fiber orientation. Indeed, a recent effort to follow the full trajectory of this bundle was unsuccessful^25^.

The transverse fasciculus of the lingual lobule of Vialet and TFC are described by the Dejerines as having opposing origins along the calcarine fissures and mirroring trajectories. This implies the possibility of complementary functions related to the upper and lower quadrants of the visual field, and integration of visual stimuli to the accessory visual integration area and the rest of the cerebrum^45,46^. It follows that if the transverse fasciculus of Vialet exists to connect the areas of the cortex involved in the superior visual fields with distal cortical regions, a similar subcortical system would need to be in place for the inferior visual fields.

### The sledge runner fasciculus (SRF)

The SRF had not been described by the Dejerines or other neuroanatomists until Vergani and colleagues^25^. The tract was initially named the ‘sledge-runner fasciculus’ to highlight its undulating shape due to its anteriorly projecting curve at the level of the calcarine fissure^25^. Later, in a focused tractography study, the SRF was alternatively named as the Medial Occipital Longitudinal Tract (MOLT) to better reflect its anatomy^64^. We continue to use the term SRF as it is the more widely known term. The SRF is a thin tract that lies within the medial aspect of the anterior calcarine fissure relaying the anterior border of the dorsal occipital lobe to the antero-superior aspect of the lingual gyrus (Fig. 5). Previous tractography studies align with our observation that the SRF lies deep to the anterior third of the stratum calcarinum (Fig. 6). The SRF connects the anterior border of the medial cuneus to the lingula, isthmus of the cingulum and posterior parahippocampal gyrus^20,25,41^. Vergani et al.^25^, supposed that previous anatomists failed to identify the SRF because they classically examined the brain using coronal sections. This allowed U-fibers, running in the coronal plane along the medial and inferior aspects of the hemisphere, to mask the tract. Subsequently, studies combining both postmortem dissections and tractography confirmed the dorsomedial-ventrolateral trajectory of the SRF, specifying that it connects the posterior part of the precuneus to the lingual gyrus and that it lies predominantly medial to the occipital horn and the posterior two-thirds of the atrium^20,41^. The SRF abuts the posterior cingulum and passes medial to the forceps major with a convex anterior projection.

Furthermore, based on current functional understanding of the regions near the SRF tract dorsal origins, specifically the posterior precuneus, it is postulated that the SRF has a role in processing visual stimuli for visuospatial attention and the recognition of places^20,41^. The present study is in concordance with past findings and shows the SRF’s relations to other intraoccipital tracts (Fig. 6).

### Limitations

As we have shown throughout this study, dMRI has permitted the explorative and comparative studies of subcortical architecture and morphology in large pools of healthy patients, producing both qualitative and quantifiable in vivo data. Regardless, it is necessary to acknowledge the inherent limitations of dMRI based tractography and the specific shortcomings of the technique used in our study. First, it is well documented that the foundation of all successful white matter studies is a strong anatomical background to limit tract misinterpretation and erroneous definitions^58,65^. Interpretation of the results depends on the expertise of the operator in both physical and virtual dissections. Second, literature has extensively noted that tractography struggles to discern crossing and kissing fibers^58^. Third, the inaccurate localisation of tract endpoints is a persistent issue despite the improvement in image resolution^66,67^. Identification of endpoints requires heuristic selection techniques and uses varied termination criteria, introducing a considerable operator bias. Fourth, selecting a method that prevents spurious inferences, by limiting the trade-off between sensitivity and specificity, when reconstructing tracts is an unsolved problem^65,68^. Furthermore, the anatomical accuracy of tractography relies on the selected algorithm parameters^65,69^, with their optimisation varying from bundle to bundle. The choice of these parameters can lead to biased characterisation of connectome topography and therefore remains a source of controversy.

One of the standard approaches to try to reduce the spurious results of tractography, particularly false positives, is the implementation of anatomical knowledge into the interpretation of the results. A form of anatomical confirmation is invasive postmortem human brain dissection. This study consulted the work of classical neuroanatomists, such as the Dejerines, to guide in vivo dissections.

With the growing sparsity of appropriate brain cadavers, the development of tractography has used the historic work of past neuroanatomists to supplement their imaging findings^26,54,70–72^. The 19th-century neuroanatomists were masterful in their blunt postmortem dissections and histological staining studies. Nevertheless, the Dejerines, and the other neuroanatomists they reviewed in their work, used brains that were not necessarily healthy, did not use the Klingler’s technique, nor was there a clear consensus on the definition of association fibers. The first two likely led to artefacts and unclear structures, and the latter incorporated a bias into the interpretation of their findings. There are therefore limits to using historical dissection work to guide new findings. This is especially the case since different neuroanatomists omitted certain tracts from their atlases that they judged, rightly or wrongly, to be inaccurate. Historical work has proven to be a useful starting point for reviving unexploited concepts. It also serves as a strong guideline in helping to determine the potential value of a study. Regardless, they do not circumvent the need for subsequent validation from the modern postmortem dissection literature.

Tractography has generated findings shrouded in controversies, many dating from the 19th century that are still unresolved. Yet, great strides have been made in acknowledging and reducing biases. In this study we have shown that there is a concordance between the original anatomical work of the Dejerines and the recently well-defined VOF, giving us confidence to extrapolate trust in our findings of the other lesser-known tracts. The results indicate gaps in current knowledge and encourage future studies based on this perspective of what the association fibers of the occipital lobe entail.

## Methods

The study consisted of two principal methods: the first being the translation from French into English of the Dejerines’ classical study of occipital intralobar fibers. This was followed by a diffusion MRI (dMRI) based virtual reconstruction of the Dejerines’ dissection in healthy subjects.

### Translation

The masterful description of intraoccipital fiber tracts in Chapter 5: pp. 780–784 by J.J. Dejerine and A. Dejerine-Klumpke in *Anatomie Centre Nerveux; Volume I* (1895) was fully translated (MB) from its original 19th-century French to more universally accessible English and reviewed for its accuracy by the experts in this field (CB, MTS). The complete translation is available as a Supplementary note.

### Subject selection

A template-based, probabilistic, fiber tractography study was conducted in 24 healthy and unrelated consenting volunteers (range 22–35 years, 12 males). The analysed diffusion MRI dataset was derived from the S1200 release of the publicly available WU-Minn Human Connectome Project (HCP) database (https://db.humanconnectome.org/). To ensure data homogeneity, the subjects selected had none of the following features/criteria: endocrine abnormalities including hyper and hypothyroidism, a handedness score below zero (people with a left-handed tendency), color vision abnormalities, illegal drug use, history of psychiatric problems, hypertensive individuals, alcohol detected by a breathalyser, data with quality control (QC) issues A or B. This study uses data collected and processed by the HCP and approved by the Washington University IRB. The additional data analysis conducted in this present study was approved by the local ethics committee of the University of Malta.

### Image acquisition

The full details of image acquisition can be found in the following articles^73–77^. In short, the HCP group generated the in vivo T1-weighted and diffusion weighted magnetic resonance imaging (MRI) of the human brains through a customised high-resolution 3-Tesla Siemens Skyra scanner equipped with a 32-channel head coil. Structural imaging utilised an axial 3D MPRAGE pulse sequence, while the dMRI used a monopolar Sejskal-Tanner sequence^78,79^. Following the algorithm in ref. ^80^ DICOM image files were defaced and deidentified. For further data analysis, dcm2nii MRIcron was used to convert DICOM files to nifti^81^ (http://www.nitrc.org/projects/mricron).

Diffusion data were rectified for head motion and geometrical distortion, and preprocessed through the HCP minimally processed pipeline^15,79,82–86^.

### MRI data processing

All additional processing was accomplished with the fiber tractography MRtrix3 (RC_3) software toolkit and iFOD2 algorithm^87,88^. The fiber orientation distribution (FOD) functions from the diffusion signal in each voxel were computed using the multi-shell-multi-tissue CSD.

The individual FODs across the 24 subjects were used to create a population template within MRtrix3. A manually defined occipital lobe mask on the template was then used to produce occipital lobe specific connectomes. The safety margins between the occipital lobe and the exclusion border were large and included posterior aspects of the temporal and parietal lobe. The mask excluded the brainstem, the cerebellum. The anterior border of the mask was defined by a coronal line at the anterior aspect of the splenium of the corpus callosum that extended straight to the lateral convexity of the cerebrum. Figures of the mask are provided in https://osf.io/5v92x/. The connectome derived from 10 million randomly seeded fibers from within it (iFOD2, Lmax = 8, Length: 10–150 mm, Max angle = 45 degrees, output step size = 0.625 mm, FOD cutoff = 0.05, FOD power = 3)^89,90^. Fibers traversing beyond the occipital lobe were manually assessed. Inclusion and exclusion masks are available through the osf site. Each ROI is titled in a way that reflects the order in which it was applied with a number, the name of tract it is identifying, and in which hemisphere. The first ROI selected was always an inclusion ROI to reduce the bulk of the tract and narrow it around an anatomical landmark as described in the work of the Dejerines. Within the convention of the MRtrix3, inclusion ROIs retain any tracts passing through the MRI mask regardless of their origin and termination. Inclusion ROIs were generally followed by exclusions to remove spurious fibers.

Following template space dissections, the process was repeated in all 24 individual subjects where all masks and ROIs defined in template space were warped into native space (with linear interpolation followed by a binarization with no thresholding to overestimate the native regions).

### Reporting summary

Further information on research design is available in the Nature Research Reporting Summary linked to this article.

## Supplementary information

Supplementary Information

Reporting Summary

## Data Availability

The datasets used and analysed in this manuscript, including results and scripts, are available in the OSF repository, https://osf.io/5v92x/. Further unprocessed, and minimally preprocessed, data can also be found through the Human Connectome Project Database, http://db.humanconnectome.org/.
