# Supplementary material for: Occipital Intralobar fasciculi: a description, through tractography, of three forgotten tracts
Source: Commun Biol. 2021 Mar 30;4:433. doi: 10.1038/s42003-021-01935-3 (PMC8010026; doi:10.1038/s42003-021-01935-3)
Supplement: Supplementary file 1 — Supplementary Information [file 42003_2021_1935_MOESM1_ESM.pdf]

SUPPLEMENTARY NOTE
Translation of Anatomy of the Central Nervous System; Section on the Intralobar Fibres (pp.780 - 786)

English Translation

Association fibres specific to the occipital lobe (*Stratum proprium cortices of Sachs). —* Fibres specific to the occipital lobe surround the inferior longitudinal fasciculus, and are arranged in five fascicles that are more or less clearly defined. These are: the *stratum calcarinum* internally, the *vertical occipital fasciculus of Wernicke*[1] externally, the *transverse fasciculus of the lingual lobule of Vialet* inferiorly, *the transverse fasciculus of the cuneus* and *the fasciculus of the cuneus of Sachs* superiorly. The vertical occipital fasciculus, the stratum calcarinum and the fasciculus of the cuneus [of Sachs] [2] are formed of fibres that are more or less vertical and link the superior part of the occipital lobe to its middle and inferior parts; the fibres of the transverse fasciculus of the cuneus and of the lingual lobule travel from the medial to lateral surface of the occipital lobe, and connect them together.

The stratum calcarinum (fig. 290 and 389, strK[3]) is a thick layer of vertical fibres that circumscribe the calcar avis[4], and separate it from the inferior longitudinal fasciculus, and independently form the white matter of the annectant cuneo-limbic gyrus. These fibres connect the upper and lower lips of the calcarine fissure ; the shortest and most superficial [fibres] join the deep parts of the two lips of the calcarine fissure; the longest fibres connect the medial surface of the cuneus to the infero-medial surface of the lingual lobule. This layer of vertical fibres extends from the occipital pole to the limbic lobe. They unite at the common branch of the calcarine and parieto-occipital fissures, the second limbic gyrus (hippocampal gyrus) and the annectant retro-limbic gyrus of Broca[5]; in this region they reinforce the posterior fascicle of the cingulum. In other words, they represent a U-fibre layer of the calcarine fissure.

Brissaud describes the stratum calcarinum as the *scalloped blade of the cuneus*[6], and he supports the hypothesis - substantiated through the macroscopic examination of

---

[1] “The names referred to throughout the chapter are the researchers whose work was under discussion at the time and were cited by the Dejerines”
[2] Original: le faisceau propre du cunéus
[3] “The letters appearing throughout the chapter refer to the labels found in the original diagrams.”
[4] Original: qui double l'écorce de l’ergot de Morand
[5] Original: le pli retro-limbique de Broca.
[6] Original: lame festonnée du cunéus

normal brains fixed in the bichromate -that the stratum calcarinum[7] completely isolates the cuneus from the rest of the hemisphere, and that it opposes a barrier to the projection fibres, of which the cuneus would thus be deprived[8]. Microscopic examination of occipital lobe sections, stained by the Weigert or Pal methods, shows how inadequate the macroscopic sections are for determining the path of a bundle. Here, as in all the other regions of the cortex, we see fibres radiating from the cortex of the calcarine fissure crossing the layer of U-shaped fibres perpendicularly, or somewhat obliquely, and then [crossing] the association fibres of varying length, to contribute to the layer of the projection fibres, which are always situated deep and close to the ventricular cavities.

The systematic study of the limited lesions of the cuneus and the degeneration that they cause shows that (v. Monakow, Moeli, Henschen, Zinn, Vialet) the cuneus has projection fibres just like the other regions of the cerebral cortex, and that these fibres arrive at their destination by the shortest path, that is to say, by crossing the U-shaped fibres and the association fibres of varying length, before arriving to the layer of sagittal fibres of the occipital lobe .

The vertical occipital fasciculus or perpendicular occipital fasciculus of Wernicke (Ov), (fig. 377, 388, 389), *stratum proprium convexitatis of Sachs*, is a thick layer of fibres specific to the occipital lobe which connects the superior edge of this lobe to its inferior surface. It therefore connects the superior occipital gyrus[9] with the inferior occipital gyrus[10], this layer thins anteriorly; it connects the angular gyrus[11] to the middle and inferior temporal gyri[12] and thickly covers the thin layer of the fibres belonging to the parallel sulcus.

Behind the supramarginal gyrus, the vertical occipital fasciculus of Wernicke merges with the posterior or descending fibres of the superior longitudinal or arcuate fasciculus of Burdach.

---

[7] Original: cette lame festonée

[8] Original: “Brissaud décrit le stratum calcarinum sous le nom de lame festonnée du cunéus et soutient l'hypothèse - étayée sur l’examen macroscopique de cerveaux normaux durcis dans le bichromate, - que cette lame festonnée isole complètement le cunéus du reste de l'hémisphère, et qu’elle oppose une barrière aux fibres de projection dont le cuneus serait ainsi dépourvu.” - We interpret this to mean that Brissaud thought that the stratum calcarinum creates a barrier to projection fibres, however, we note that the text in this instance is not entirely clear.

[9] Referred to by the Dejerines as the ‘first occipital gyrus’

[10] Referred to by the Dejerines as the ‘third occipital gyrus’

[11] Original: le pli courbe

[12] Referred to by the Dejerines as the ‘second and third temporal gyri’

As a whole, the fibres of the occipito-frontal fasciculus[13] thus constitute a kind of vertical septum extending from the occipital pole to the posterior branch of the Sylvian fissure. This vertical septum is traversed by the numerous fibres that enter into inferior longitudinal fasciculus, optic radiations[14] of the occipital-temporal lobe and the tapetum ; it is further crossed by the fibres of the transverse fasciculus of cuneus and the lingual lobule. Due to these numerous intersections, this layer of vertical fibres is poorly known laterally, it is on the contrary clearly delimited medially by the inferior longitudinal fasciculus, and its fibres are distinguished from those of the latter by their direction and their clearer coloring by hematoxylin stain.

The transverse occipital fasciculus of the cuneus[15] (*stratum cunei transversum of Sachs) (*ftcS) (fig. 290, 384 and 389) links the cuneus to the convexity of the occipital lobe and to its infero-lateral border; it belongs to the region of the cuneus, does not protrude past the parieto-occipital fissure, and has the same cortical origin as the stratum calcarinum. Its fibres arise from the upper lip of the calcarine fissure and project transversely and laterally; but instead of bending downwards like the fibres of the stratum calcarinum, they curl upwards, pass over the hollow cone formed by the occipital part of the inferior longitudinal fasciculus ,then pass through the vertical occipital fasciculus, intertwine with the commissural projection and association fibres of the region, and most likely radiate in the cortex of the convexity of the occipital lobe and its infero-lateral border. The most anterior fibres run a slightly obliquely, anteriorly and laterally[16], and radiate in the superior parietal lobule and in the angular gyrus[17] (Sachs).

The transverse fasciculus of the lingual lobule of Vialet (fig. 290 and 389, ftlgV) is to the lingual lobule what the previous fasciculus to the cuneus. Glimpsed by Sachs, well studied by Vialet, this fascicle is born from the lower lip of the calcarine fissure, projects transversely and laterally, and then inferiorly overlaps with the inferior longitudinal fasciculus. Its fibres are reflected at the level of the diverticulum of the lingual lobule and the medial basal bundle of Burdach, and a second time at the infero-lateral angle of the occipital horn, then they cross the vertical occipital fasciculus of Wernicke, and radiate into the cortex of the convexity of the occipital lobe and its infero-lateral border. This fascicle connects the lower lip of the calcarine fissure to the convexity of the hemisphere. According to Vialet it represents the lower half of the association system that connects the calcarine region to the occipital convexity. In certain lesions of the occipital lobe it is sometimes found intact, among the degenerated

---

[13] Note that this is not the inferior-fronto-occipital fasciculus that dominates the modern literature but a dorsal pathway that is sometimes referred to as the superior fronto-occipital fasciculus.
[14] Referred to by the Dejerines as the ‘Thalamic radiations’
[15] This a fasciculus which was omitted (perhaps erroneously) from original five fasciculus they list at the start of this chapter.
[16] Original: se portent un peu obliquement en avant et en dehors
[17] Original: le pli courbe

fibres of the region, and these cases are particularly instructive for the study of this fascicle (fig. 394).

In addition to these four layers of occipital lobe fibres, Sachs described a fifth layer of short association fibres specific to the cuneus, it is the stratum proprium cunei of Sachs (fig. 309. strprC). These are also vertical fibres like those of the stratum calcarinum: they originate similarly to those of the previously mentioned layer and like those of the transverse fasciculus of the cuneus in the upper lip of the calcarine fissure, then projecting dorsally, overlie the fibres belonging to the sulci of the cuneus and radiate into the cortical region of the superior border of the hemisphere.

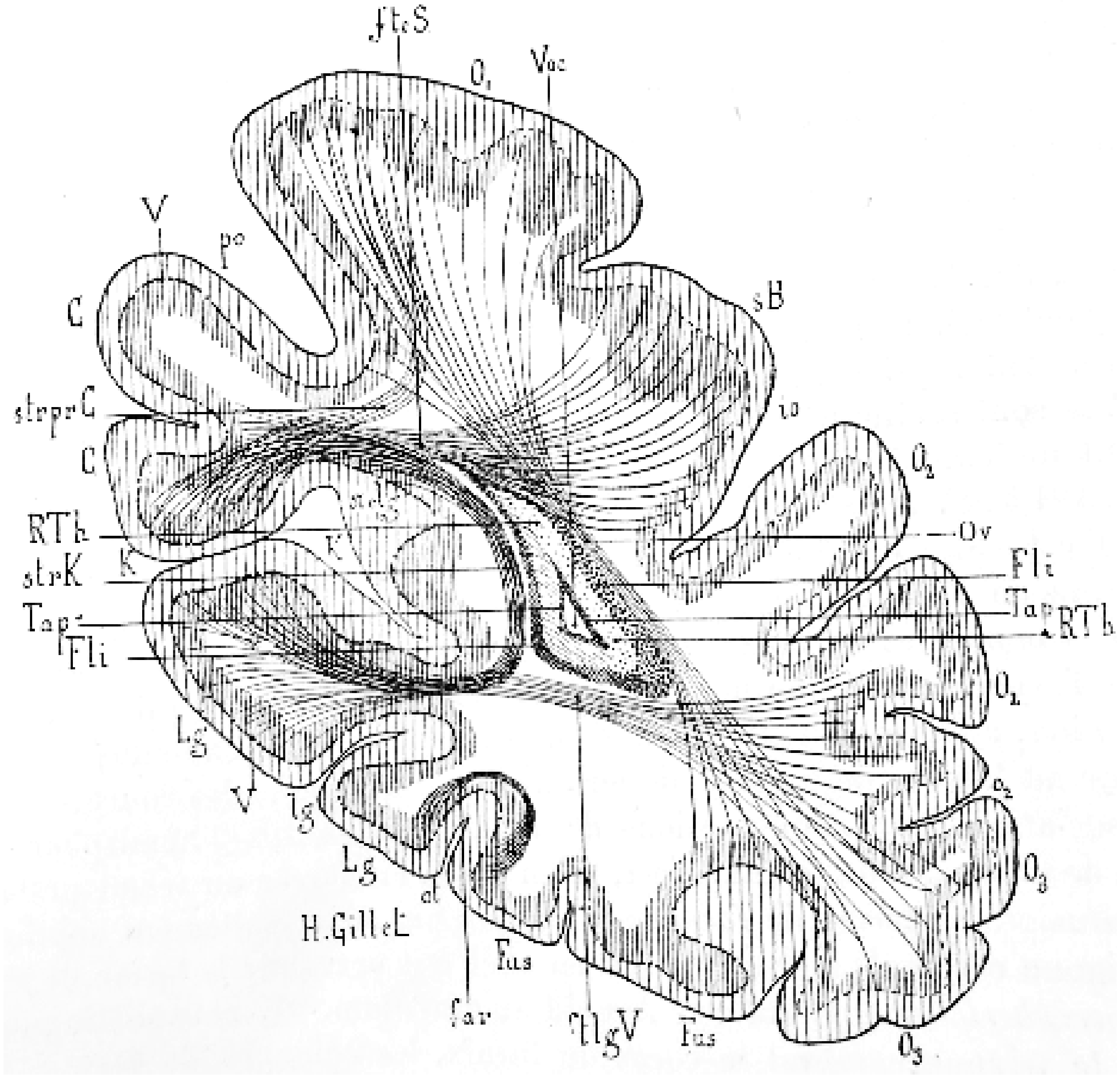

Fig. 389. — Vertico-transverse cross section of the left occipital lobe, intended to show the origin and path of the association fibres of the occipital lobe. (Half schematic.)
C, cuneus. — *far*, arcuate fibres. — *Fli*, inferior longitudinal fasciculus. — *fllg V,* transverse fasciculus of the lingual lobule of Vialet. — *ftcS*, transverse fasciculus of the cuneus. —
Fus, fusiforme lobule. — io, inter-occipital sulcus. — K, calcarine sulcus. — Lg, ligual gyrus.— lg, ligual lobe sulcus. — 01, 02, 03, first, second and third occipital gyri. — *o2*, second occipital sulcus. — *Ov*, vertical occipital fasciculus. —*ot* collateral sulcus. — *po*,parieto-occipital sulcus. — *nclg*, cuneo-limbic fold. — *RTh*, Gratiolet radiation. — *sB*, Line of Baillarger. — *strK,* stratum calcarinum. —
slrprC, stratum proprium cunei. — *Tap*, tapetum. — *V*, line of Vicq d'Azyr. — *Voe*, occipital horn of the lateral ventricle.

The layer of fibres specific to the gyri of the medial surface of the hemisphere. — A layer of vertical fibres, similar to the *fibres of the cuneus*, is also found in the *precuneus*, in the *precentral lobule* and in the *medial surface* of the *first frontal gyrus*. These fibres, of varied length, arise from the upper edge of the hemisphere, project obliquely inferiorly and anteriorly, and terminate around the calloso-marginal and subparietal fissures. This layer of fibres extends from the cuneus to the frontal pole, and intersects with the numerous projection and commissural fibres which approach the gyri of the medial surface of the hemisphere; thanks to the oblique direction of its fibres, this layer appears darker on the vertico-transverse sections of brains hardened in the dichromate (fig. 239 to 260), and is therefore easily distinguished from callosal radiations and fibres of the corona radiata, which circumvent the roof of the lateral ventricle and whose fibres are cut parallel to their length, as clearly shown by the microscopic vertical-transverse sections.

It is this layer of fibres specific to the gyri of the medial surface of the hemisphere, which Brissaud designated by the name of *compact fascicle* and *diffuse fascicle of the fornix*. There can be no question in this case, as we have just seen, of a long association fibre, analogous to the cingulum and extending from the frontal pole to the cuneus, or even to the lingual gyrus. To apply the name of “tracts of the fornix” to the layer of short association fibres of the gyri of the medial surface of the hemisphere is, in our opinion, to use a confusing term. The term *fornix* is today universally applied to the cerebral trigone; its use prevented the term *peripheral fornix,* applied by Arnold to the cingulum, from prevailing. Yet, we distinguish in the cerebral trigone the body of the fornix, the columns of the fornix, the pillars of the fornix; the works of Gudden, Forel, Honegger, etc., have shown that the cerebral trigone contains a very complex system of fibres: in addition to the fornix itself, there is described a *fornix transversus*, a *fornix longus* direct and crossed, a *fornix obliquas*, ect. For all these reasons, we will therefore reserve the term of *fornix* for the cerebral trigone and designate the layer of association fibres referred to here as “*the layer of fibres specific to the gyri of the medial surface of the hemisphere”.*

In the **frontal lobe**, the system of specific association fibres is much less developed than in the occipital lobe. The occipital horn, around which the sagittal layers and the different layers of the short association fibres are grouped, extends far into the occipital lobe, while the frontal horn hardly extends beyond the anterior extremity of the caudate nucleus. Nevertheless, on the coronal sections, immediately anterior to of the head of the caudate nucleus and around the subependymal gray matter (Sge), which circumscribes the frontal horn (Vf) anteriorly, an annular layer arrangement, quite similar in appearance, though much reduced, to what is observed in the occipital horn (Fig. 390). The inner ring is formed within, above and below by the callosal fibres emanating from the genu (Ce), it is completed outside by the occipito-frontal fasciculus (OF). Around this first system of fibres, there is the irregular and incomplete ring formed by the fibres of the corona radiata of the frontal lobe (CR). These fibres are arranged in a thick layer laterally, superiorly and inferiorly, and thin medially, where they are reinforced by the fibres of the cingulum, immediately, anterior to the genu of the corpus callosum. The fibres of the orbital and outer portions of the corona radiata are sectioned perpendicular to their length. The fibres of the upper parts of the corona radiata are cut more or less obliquely or parallel to their axis, they radiate towards, in fact, the medial and superior surfaces of the first frontal gyrus and in the cingulate gyrus[18] (Fig. 390).

This arrangement in sagittal layers is not found on horizontal sections (Fig. 391, 296), the latter show, on the contrary, that the callous fibres are reflected anteriorly to the lateral ventricle before radiating towards the third frontal gyrus, and that the fibres of the corona radiata suggest a curve in the opposite direction around the callous fibres. The arrangement in concentric rings is due only to a false appearance, obtained thanks to the simultaneous section of the fibres of the genu of the corpus callosum and its radiating fibres.

It is around the fibres of the corona radiata that the layers of association fibres specific to the frontal lobe are grouped: some take a transverse direction and connect the medial surface of the frontal lobe to its orbital and lateral surfaces; the others have a vertical direction and provide the connections, either between the different gyri of its medial surface, or between the gyri of its orbital and super-external sides. Lastly, others affect in a sagittal direction; they are particularly numerous in front of the anterior perforated space and intersect with the anterior extremities of the fibres of the uncinate fasciculus, which radiate in the orbital surfaces of the first and third frontal gyri.

But these short association fibres are not arranged in compact layers, as in the occipital lobe — they intersect with the numerous projection and commissural fibres of the region. In front of the subependymal gray matter, the layers of differentiated fibre

[18] Referred to by the Dejerines as ‘the first limbic gyrus’.

rapidly lose their individuality, such that most of the white matter of the frontal lobe is formed by the intimate entanglement of the association fibres with the commissural and projection fibres. It is along the olfactory sulcus, in the gyrus rectus and the orbital part of the first frontal gyrus, that we can best follow the aforementioned fibres (Figs 242 and 243, pp. 442 and 445).

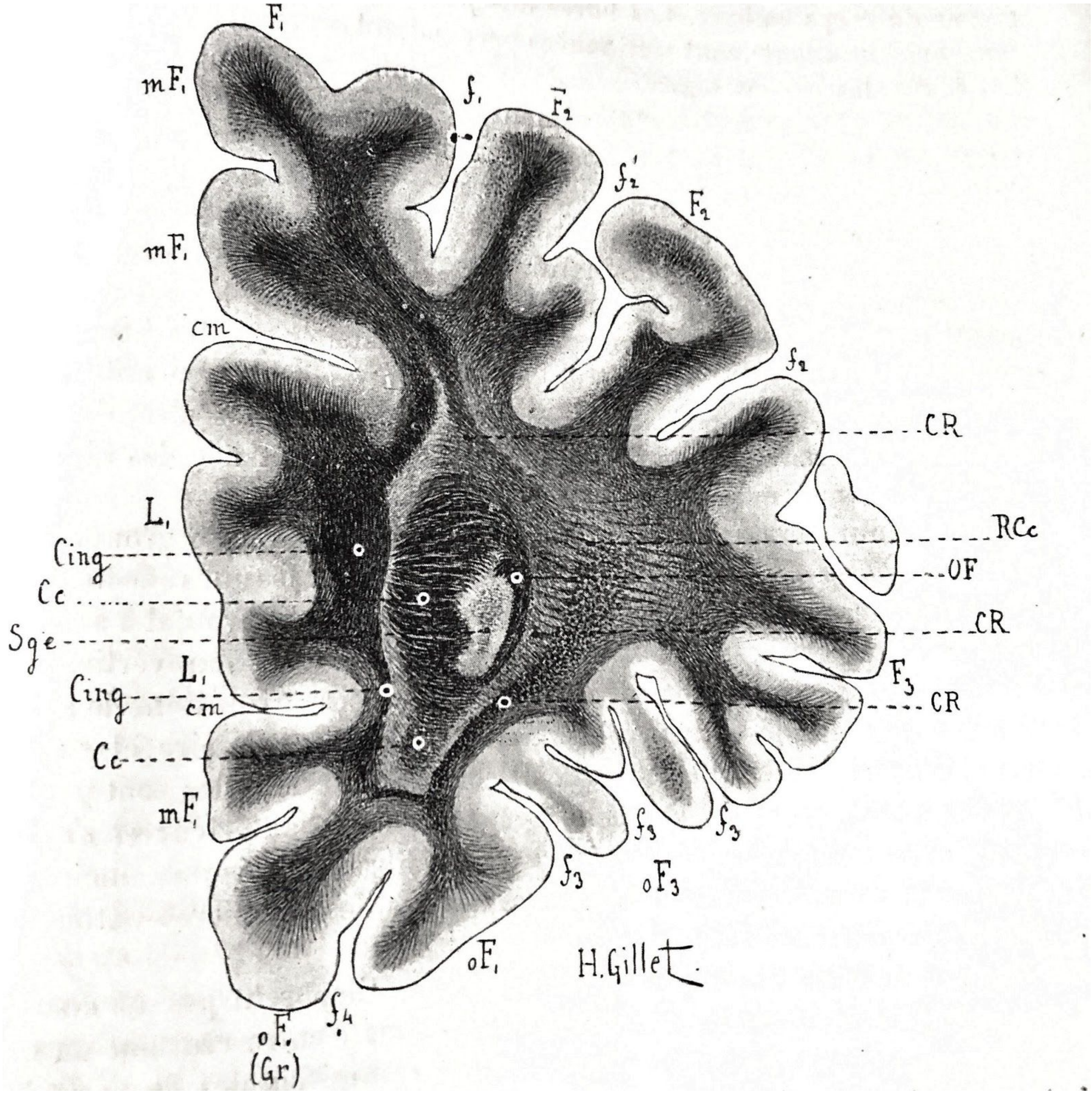

Fig. 390. Vertico-transverse cross-section of the right hemisphere, cutting in front of the genu of the corpus callosum and of the frontal horn of the lateral ventricle. The Weigert method. Enlargement of 3/2. Details drawn to an enlargement of 12 diameters. (See, for the description of this cross-section, p. 321, cross-section n°1.)

Cc, callosal fibres. — *Cing,* cingulum. — *cm,* calloso-marginal sulcus. — *CR,* corona radiata. — F_1, F_2, F_3, first, second and third fronal gyri. — f_1, f_2, f_3, first, second and third frontal sulci. — f_1, olfactory sulcus. —f_2, second frontal sulcus. — L_1, cingulate gyrus. — mF_1, medial surface of the first frontal gyrus. — *OF*, occipito-frontal fasciculus. oF_1, orbital portion of the cingulate gyrus. — $oF_1(Gr)$, orbital portion of the cingulate gyrus (rectus gyrus).— oF_3, orbital portion of the third frontal gyrus. — *RCc*, optic radiations of the corpus callosum. — *Sge*, subependymal gray matter.

Original French Text

Fibres d’association propres au lobe occipital *(Stratum proprium corticis de Sachs)*. - Autour du faisceaux longitudinal inférieur se groupent *les fibres propes au lobe occipital*, disposées en cinq faisceaux plus ou moins nettement délimités. Ce sont: en dedans le *stratum calcarinum*, en dehors le *faisceau occipital vertical* de Wernicke, en bas le *faisceau transverse au lobule lingual de* Vialet, en haut le *faisceau transverse du cunéus* et le *faisceau propre du cunéus* de Sachs. Le faisceau occipital vertical, le stratum calcarinum et le faisceau propre du cunéus sont formés de fibres plus ou moin verticales, qui relient la partie supérieure du lobe occipital à ses parties moyenne et inférieure ; les fibres de faisceaux transverses du cunéus et du lobule lingual se dirigent de dedans en dehors, et assurent les connexion entre les faces interne et externe de ce lobe.

Le stratum calcarinum (fig. 290 et 389, strK) est une épaisse couche de fibres verticales, qui double l'écorce de l’ergot de Morand, la sépare du faisceau longitudinal inférieur et forme à elle seule la masse blanche des plis cunéo-limbiques. Ses fibres relient la lèvres supérieure de la scissure calcarine à sa lèvre inférieure; les plus courtes et les plus superficielle unissent les partie profondes des deux lèvres de la scissure calcarine, les fibres les plus longues relient la face interne du cunéus à la face inféro-interne du lobule lingual. Cette couche de fibres verticales s'étend du pôle occipital au lobe limbique, unit au niveau de la branche commune aux scissures calcarine et pariéto-occipitale, la deuxième circonvolution limbique (circonvolution de l’hippocampe) au pli rétro-limbique de Broca, et renforce dans cette région le faisceau postérieur du cingulum. Elle représente en d’autres termes, la couche des fibres en U de la scissure calcarine.

Brissaud décrit le stratum calcarinum sous le nom de *lame festonnée du cunéus* et soutient l'hypothèse - étayée sur l’examen macroscopique de cerveaux normaux durcis dans le bichromate, - que cette lame festonnée isole complètement le cunéus du reste de l'hémisphère, et qu’elle oppose une barrière aux fibres de projection dont le cuneus serait ainsi dépourvu. L’examen microscopique de coupes du lobe occipital, colorées par le methodes de Weigert ou de Pal, montre combien les coupes macroscopique sont insuffisant pour déterminer le trajet d’un faisceau ; ici comme dans

toutes les autres région de l'écorce, on voit les fibres radiées de l'écorce de la scissure calcarine, traverser perpendiculairement ou plus ou moin obliquement la couche des fibres en U, puis les faisceaux d'association plus ou moins longs, et concourir à former la couche des fibres de projection, dont la situation est toujour profonde et voisine des cavités ventriculaires.

L'etude systematique des lésions limitées du cuneus et des dégénérescences qu'elles entraînent montre du reste (v. Monakow, Moeli, Henschen, Zinn, Vialet), que le cunéus possède des fibres de projection tout comme les autres région de l'écorce cérébrale, et que ces fibres arrivent à leur destination par le plus court chemin, c'est-à-dire en traversant les fibres en U et les fibres d'association plus ou moins longues, avant d'arriver à la couche des fibres sagittales du lobe occipital.

Le faisceau occipital vertical ou **occipital perpendiculaire** de Wernicke (Ov), (fig. 377, 388, 389) *stratum proprium convexités de Sachs*, constitue une épaisse couche de fibres propres au lobe occipital et qui relie le bord supérieur de ce lobe a sa face inférieure. Il met par conséquent en connexion la première circonvolution occipital avec la troisième circonvolution occipital, cette couche s'amincit en avant ; elle relie le pli courbe aux deuxième et troisième circonvolution temporales et recouvre profondément la mince couche des fibres propres du sillon parallèle.

En arrière du gyrus supra-marginalis, le faisceau occipital vertical de Wernicke se confond avec les fibres postérieures ou descendantes du faisceau arqué ou longitudinal supérieur de Burdach.

Dans leur ensemble, les fibres du faisceau occipito-frontal constituent donc une sorte de cloison verticale, étendue de la pointe occipital à la branche postérieure de la scissure de Sylvius. Cette cloison est traversée par les nombreuses fibres qui entrent dans la constitution du faisceau longitudinal inférieur, des radiations thalamiques du lobe occipito-temporal et du tapetum ; elle est encore traversée par les fibres des faisceaux transverse du cuneus et du lobule lingual. Grace a ces nombreux entre-croisements, cette couche de fibres verticales est mal délimitée en dehors, elle est au contraire nettement délimitée en dedans par le faisceau longitudinal inférieur, et ses fibres se distinguent, de celles de ce dernier par leur direction et leur coloration moins foncée par la laque hématoxylinique.

Le **faisceau occipital transverse du cunéus** (*stratum cunei tranversum de Sachs*) (ftcS), (fig.290, 384 et 389) relie le cunéus à la convexité du lobe occipital et à son bord inféro-externe ; il appartient à la région du cunéus, ne dépasse pas en avant la scissure pariéto-occipitale, et présente la même origine corticale que le stratum calcarinum. Ses fibres naissent donc de la lèvre supérieure de la scissure calcarine et se portent transversalement en dehors; mais au lieu de s'infléchir en bas comme les fibres du stratum calcarinum, elles se recourbent en haut, passent au-dessus du cône

creux formé par la partie occipitale du faisceau longitudinal inférieur, puis traversent le faisceau occipital vertical, se mélangent intimement aux fibres commissurales de projection et d'association de la région, et s'irradient très probablement dans l'écorce de la convexité du lobe occipital et de son bord inféro-externe. Les fibres les plus antérieures se portent un peu obliquement en avant et en dehors, et s'irradient dans le lobule parietal supérieur et dans le pli courbe (Sachs).

Le faisceau occipital transverse du lobule lingual de Vialet (fig. 290 et 389, AlgV) est au lobule lingual ce que le faisceau précédent est au cunéus. Entrevu par Sachs, bien étudié par Yialet, ce faisceau naît delà lèvre inférieure de la scissure calcarine, se porte transversalement en dehors, puis recouvre en bas le faisceau longitudinal inférieur qu'il tapisse; ses fibres se réfléchissent une première fois au niveau du diverticule du lobule lingual et du faisceau basal interne de Burdach, une seconde fois au niveau de l'angle inféro-externe de la corne occipitale, puis elles traversent le faisceau occipital vertical de Wernicke, et s'irradiant dans l'écorce de la convexité du lobe occipital et de son bord inféro-externe.Ce faisceau, qui relie la lèvre inférieure de la scissure calcarine à la convexité de l'hémisphère, et qui représente, d'après Vialet, la moitié inférieure du système d'association qui met en relation la région calcarinienne avec la convexité occipitale, se trouve quelquefois, dans certaines lésions du lobe occipital, persister intact, parmi les fibres dégénérées de la région, et ces cas sont particulièrement instructifs pour l'étude do ce faisceau (fig. 394)(Voy.Th.de Yialel. Obs. III).

Outre ces quatre couches de fibres propres au lobe occipital, Sachs décrit une cinquième couche de courtes fibres d'association propres au cunéus, c'est le stratum proprium cunei de Sachs (fig. 309. strprC). Il s'agit ici encore de fibres verticales comme celles du stratum calcarinum : elles prennent leur origine comme celles de cette dernière couche et comme celles du faisceau transverse du cunéus, dans la lèvre supérieure de la scissure calcarine, puis se portent verticalement en haut, recouvrent les fibres propres des sillons du cunéus et s'irradiant dans l'écorce du bord supérieur de l'hémisphère.

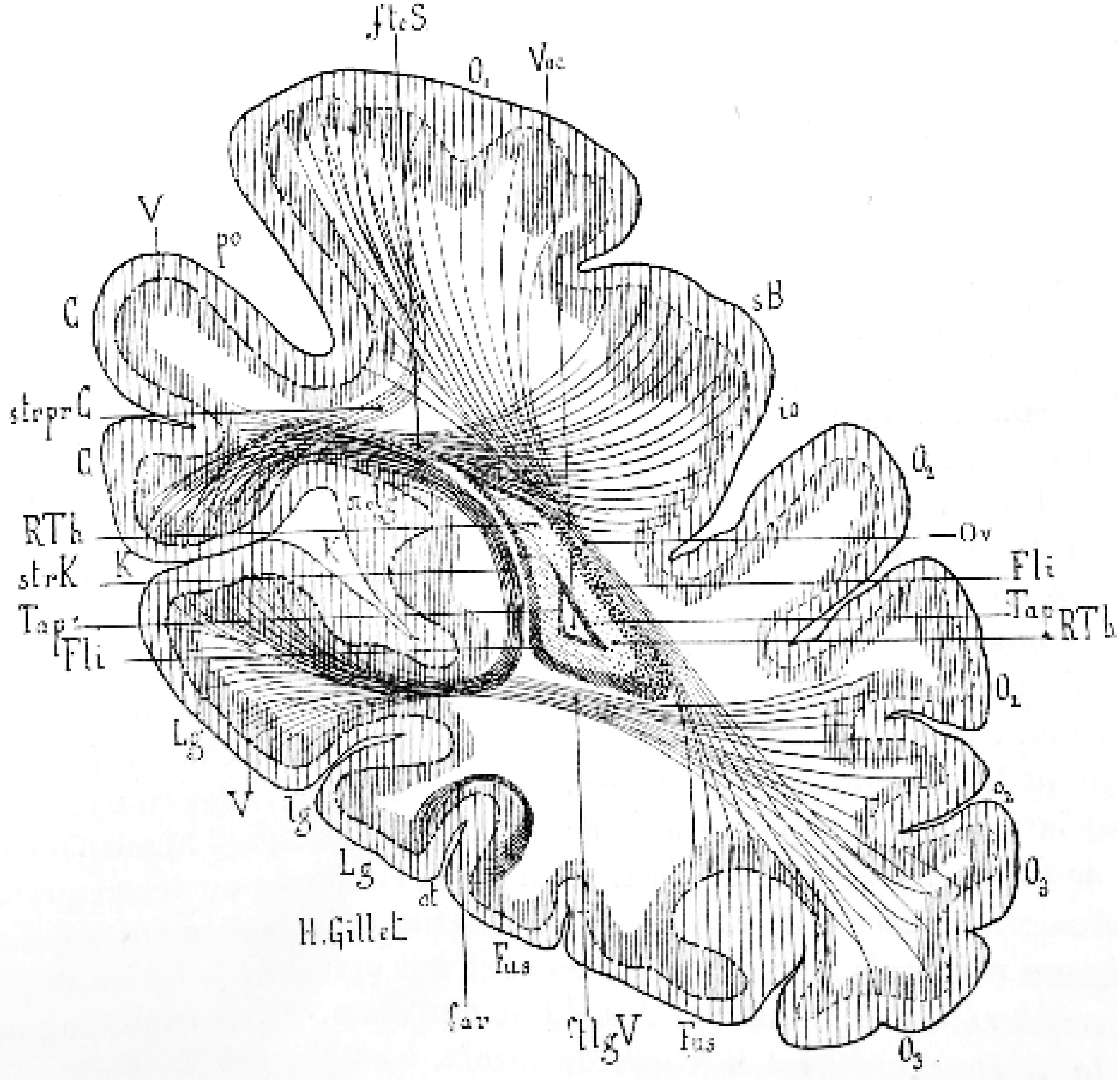

Fig. 389. — Coupe vertico-transversale du lobe occipital gauche, destinée à montrer l'origine et le trajet des fibres d'association propres du lobe occipital. (Demi-schématique.)

C, cunéus. — far, fibres arquées. — Fli, faisceau longitudinal inférieur. — fllg V, faisceau transverse du lobule lingual de Vialet. — ftcS, faisceau transverse du cunéus de Sachs. — Fus, lobule fusiforme. — io, sillon inter-occipital. — K, scissure calcarine. — Lg, lobule lingual.— lg, sillon du lobule lingual. — 01, 02, 03, première, deuxième et troisième circonvolutions occipitales. — o2, deuxième sillon occipital. — Ov, faisceau occipital vertical. —oisillon, collatéral. — po, scissure pariéto-occipitale. — nclg, pli cunéo-limbique. — RTh, radiations thalamiques de Gratiolet. — sB, strie de Baillarger. — strK, stratum calcarinum. — slrprC, stratum proprium cunei. — Tap, tapetum. — V, ruban de Vicq d'Azyr. — Voe, corne occipitale du ventricule latéral.

Couche des fibres propres des circonvolutions de la face interne de L'hémisphère. — Une couche de fibres verticales, analogues aux *fibres propres du cunéus*, se trouve également dans le *précunéus*, dans le *lobule paracentral* et dans la face interne de la *face interne* de la *première circonvolution frontale*. Ces fibres, plus ou moins longues, naissent du bord supérieur de l'hémisphère, se portent obliquement en bas et en avant et se terminent autour des scissures calloso-marginale et sous-pariétale. Cette couche de fibres s'étend du cunéus au pôle frontal, et s'entre-croise avec les nombreuses fibres de projection et commissurales qui abordent les circonvolutions de la face interne de l'hémisphère; grâce à la direction oblique de ses fibres, cette couche apparaît foncée sur les coupes vertico-transversales de cerveaux durcis dans le bichromate (fig. 239 à 260), et se distingue par conséquent facilement des radiations calleuses et des fibres de la couronne rayonnante, qui contournent la voûte du ventricule latéral et dont les fibres sont sectionnées parallèlement à leur longueur, ainsi que le montrent nettement les coupes vertico-transversales microscopiques (p.328, fig. 281 à 287 et 387).

C'est cette couche de fibres propres des circonvolutions de la face interne de l'hémisphère, que Brissaud a désignée sous le nom de *faisceau compact* et de *faisceau diffus du fornix*. Il ne peut être question dans l'espèce, ainsi que nous venons de le voir, d'un long faisceau d'association, analogue au cingulum et étendu du pôle frontal au cunéus, voire même au lobule lingual. Appliquer en outre à la couche des courtes fibres d'association des circonvolutions de la face interne de l'hémisphère le nom de faisceaux du fornix, c'est, selon nous, employer un terme prêtant à confusion. Le terme de *fornix* est aujourd'hui universellement appliqué au trigone cérébral; l'usage n'a pas en effet fait prévaloir le terme de *fornix periphericus*, appliqué par Arnold au cingulum. Or, nous distinguons dans le trigone cérébral le corps du fornix, les colonnes du fornix, les piliers du fornix; les travaux de Gudden, Forel, Honegger, etc., ont montré en outre que le trigone cérébral renferme un système fort complexe de fibres : on y décrit, outre le fornix proprement dit, un *fornix transversus*, un *fornix longus* direct et croisé, un *fornix obliquas*, etc. Pour toutes ces raisons nous réserverons donc le terme de *fornix* au trigone cérébral et désignerons la couche de fibres d'association dont il est question ici, sous *le nom de couche des fibres propres des circonvolutions de la face interne de l'hémisphère*.

Dans le lobe frontal, le système des fibres d'association propres est beaucoup moins développé que dans le lobe occipital. La corne occipitale, autour de laquelle se groupent les couches sagittales et les différentes couches des courtes fibres d'association, se prolonge en effet très loin dans le lobe occipital, tandis que la corne frontale ne dépasse guère l'extrémité antérieure du noyau caudé. Sur les coupes vertico-transversales on constate toutefois, immédiatement en avant de la tête du noyau caudé et autour de la substance grise sous-épendymaire (Sge) qui double la

corne frontale (Vf) en avant, une disposition en couche annulaire, très analogue d'aspect, quoique beaucoup plus réduite, à ce que l'on observe dans la corne occipitale (fig. 390). L'anneau interne se trouve constitué en dedans, en haut et en bas. par les fibres calleuses émanées du genou (Ce), il est complété en dehors par le faisceau occipilo-frontal (OF). Autour de ce premier système de fibres, on trouve l'anneau irrégulier et incomplet formé par les fibres de la couronne rayonnante du lobe frontal (CR). Ces fibres se disposent en une couche épaisse en dehors, en haut et en bas, et mince en dedans, où elle est renforcée par les fibres du cingulum, immédiatement, en avant du genou du corps calleux. Les fibres des parties orbitaires et externe de la couronne rayonnante, sont sectionnées perpendiculairement à leur longueur. Les fibres des parties supérieures de la couronne rayonnante, se trouvent sectionnées plus ou moins obliquement ou parallèlement à leur axe, elles s'irradient en effet dans les faces interne et supérieure de la première circonvolution frontale et dans la première circonvolution limbique (fig. 390).

Cette disposition en couches sagittales ne se retrouve pas sur les coupes horizontales (fig. 391, 296), ces dernières montrent, au contraire, que les fibres calleuses se réfléchissent en avant du ventricule latéral avant de s'irradier dans la troisième circonvolution frontale, et que les fibres de la couronne rayonnante décrivent une courbe en sens inverse autour des fibres calleuses. La disposition en anneaux concentriques n'est due qu'à une fausse apparence, obtenue grâce à la section simultanée des fibres du genou du corps calleux et de ses fibres réfléchies.

C'est autour des fibres de la couronne rayonnante que se groupent les couches des fibres d'association propres au lobe frontal : les unes affectent une direction transversale et relient la face interne du lobe frontal à ses faces orbitaire et externe ; les autres présentent une direction verticale et assurent les connexions, soit entre les différentes circonvolutions de sa face interne, soit entre les circonvolutions de ses faces orbitaire et supéroexterne. D'autres enfin affectent une direction sagittale; elles sont particulièrement nombreuses en avant de l'espace perforé antérieur et s'entrecroisent avec les extrémités antérieures des fibres du faisceau uncinatus, qui viennent s'irradier dans les faces orbitaires des première et troisième circonvolutions frontales.

Mais ces courtes fibres d'association ne se disposent pas en couches compactes comme dans le lobe occipital — elles s'entre-croisent en effet avec les nombreuses fibres de projection et commissurales de la région. En avant de la substance grise sous-épendymaire, les couches de fibres différenciées perdent rapidement leur individualité, de telle sorte que la plus grande partie de la masse blanche du lobe frontal est formée par l'intime intrication des fibres d'association avec les fibres commissurales et de projection. C'est le long du sillon olfactif, dans le gyrus rectus et la partie orbitaire

de la première circonvolution frontale, que l'on peut le mieux suivre les dernières fibres différenciées (fig. 242 et 243, p. 442 et 445).

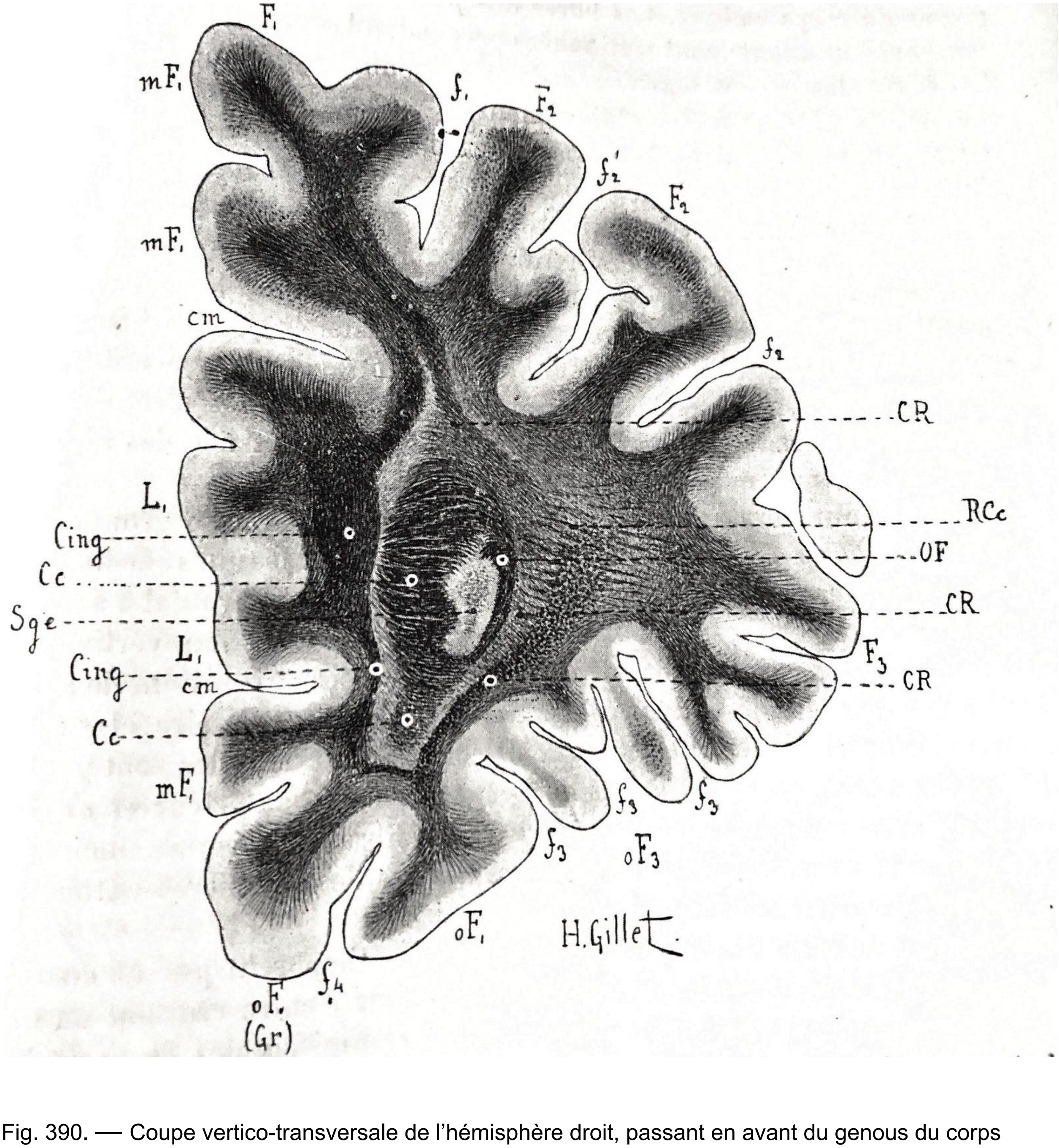

Fig. 390. — Coupe vertico-transversale de l'hémisphère droit, passant en avant du genous du corps calleux et de la corne frontale du ventricule latéral. Méthode de Weigert. Agrandissement de 3/2. Détails dessinés à un groississement de 12 diamètres. (Voy, pour la description de cette coupe, p.521, coupe n°I.)

Cc, fibres calleuses. — *Cing,* cingulum. — *cm,* sillon calloso-marginal. — *CR,* couronne rayonnante. — F_1, F_2, F_3, première, deuxième et troisième circonvolution frontales. — f_1, f_2, f_3, première, deuxième et troisième sillons fronteaux. — f_1, sillon olfactif. —f_2, incisure du deuxième convolution frontal. — L_1,

première circonvolution limbique. — *mF_1*, face interne de la première circonvolution frontal. — *OF*, faisceau occipito-frontal. — *oF_1*, partie orbitaire de la première circonvolution frontale. — *$oF_1(Gr)$*, partie orbitaire de la première circonvolution frontale (rectus gyrus) — *oF_3*, partie orbitaire de la troisième circonvolution frontale. — *RCc*, radiations du corps calleux. — *Sge*, substance grise sous-épendymaire.
