# Supplementary material for: Occipital Intralobar fasciculi: a description, through tractography, of three forgotten tracts
Source: Commun Biol. 2021 Mar 30;4:433. doi: 10.1038/s42003-021-01935-3 (PMC8010026; doi:10.1038/s42003-021-01935-3)
Supplement: Supplementary file 2 — Reporting Summary [file 42003_2021_1935_MOESM2_ESM.pdf]

nature research

Corresponding author(s): Claude J Bajada

Last updated by author(s): Jan 28, 2021

Reporting Summary

Nature Research wishes to improve the reproducibility of the work that we publish. This form provides structure for consistency and transparency in reporting. For further information on Nature Research policies, see our Editorial Policies and the Editorial Policy Checklist.

Statistics

For all statistical analyses, confirm that the following items are present in the figure legend, table legend, main text, or Methods section.

n/a	Confirmed	
☐	☒	The exact sample size (*n*) for each experimental group/condition, given as a discrete number and unit of measurement
☐	☒	A statement on whether measurements were taken from distinct samples or whether the same sample was measured repeatedly
☒	☐	The statistical test(s) used AND whether they are one- or two-sided<br>*Only common tests should be described solely by name; describe more complex techniques in the Methods section.*
☒	☐	A description of all covariates tested
☒	☐	A description of any assumptions or corrections, such as tests of normality and adjustment for multiple comparisons
☒	☐	A full description of the statistical parameters including central tendency (e.g. means) or other basic estimates (e.g. regression coefficient) AND variation (e.g. standard deviation) or associated estimates of uncertainty (e.g. confidence intervals)
☒	☐	For null hypothesis testing, the test statistic (e.g. *F*, *t*, *r*) with confidence intervals, effect sizes, degrees of freedom and *P* value noted<br>*Give P values as exact values whenever suitable.*
☒	☐	For Bayesian analysis, information on the choice of priors and Markov chain Monte Carlo settings
☒	☐	For hierarchical and complex designs, identification of the appropriate level for tests and full reporting of outcomes
☒	☐	Estimates of effect sizes (e.g. Cohen's *d*, Pearson's *r*), indicating how they were calculated

Our web collection on statistics for biologists contains articles on many of the points above.

Software and code

Policy information about availability of computer code

Data collection	All data can be found on the HCP Website: https://db.humanconnectome.org/
Data analysis	All analysis scripts can be found through this site: https://osf.io/5v92x/

For manuscripts utilizing custom algorithms or software that are central to the research but not yet described in published literature, software must be made available to editors and reviewers. We strongly encourage code deposition in a community repository (e.g. GitHub). See the Nature Research guidelines for submitting code & software for further information.

Data

Policy information about availability of data

All manuscripts must include a data availability statement. This statement should provide the following information, where applicable:

- Accession codes, unique identifiers, or web links for publicly available datasets
- A list of figures that have associated raw data
- A description of any restrictions on data availability

All results and scripts used are available on the osf website (https://osf.io/5v92x/).

Field-specific reporting

Behavioural & social sciences study design

All studies must disclose on these points even when the disclosure is negative.

Study description	A historical examination and technical reconstruction of occipital fiber tracts using diffusion tractography.
Research sample	A subsample of openly available Human Connectome Project Data
Sampling strategy	The first 24 participants who did not fit any exclusion criteria and were gender balanced. The sample size was chosen due to computational limitations. Further, this type of exploratory research likely does not need a larger sample for the claims we are making.
Data collection	Collected by the Human Connectome Project
Timing	Secondary data use
Data exclusions	The subjects selected were healthy, they had none of the following features/criteria: endocrine abnormalities including hyper and hypothyroidism, a handedness score below zero (people with a left-handed tendency), color vision abnormalities, illegal drug use, history of psychiatric problems, hypertensive individuals, alcohol detected by a breathalyser, data with quality control (QC) issues A or B.
Non-participation	NA - Secondary data use
Randomization	NA

Reporting for specific materials, systems and methods

We require information from authors about some types of materials, experimental systems and methods used in many studies. Here, indicate whether each material, system or method listed is relevant to your study. If you are not sure if a list item applies to your research, read the appropriate section before selecting a response.

Materials & experimental systems

n/a	Involved in the study	
☒	☐	Antibodies
☒	☐	Eukaryotic cell lines
☒	☐	Palaeontology and archaeology
☒	☐	Animals and other organisms
☐	☒	Human research participants
☒	☐	Clinical data
☒	☐	Dual use research of concern

Methods

n/a	Involved in the study	
☒	☐	ChIP-seq
☒	☐	Flow cytometry
☐	☒	MRI-based neuroimaging

Human research participants

Policy information about studies involving human research participants

Population characteristics	See above
Recruitment	We used the Human Connectome Project Database. Considering the type of research this is not likely to have caused any problems. The data is high quality.
Ethics oversight	This study uses data collected and processed by the HCP and approved by the Washington University IRB. The additional data analysis conducted in this present study was approved by the local ethics committee of the University of Malta.

Note that full information on the approval of the study protocol must also be provided in the manuscript.

Magnetic resonance imaging

Experimental design

Design type	Structural study
Design specifications	No experiment
Behavioral performance measures	NA

Acquisition

Imaging type(s)	Structural and diffusion
Field strength	3T
Sequence & imaging parameters	All details can be found here: http://protocols.humanconnectome.org/HCP/3T/imaging-protocols.html
Area of acquisition	Whole brain

Diffusion MRI ☒ Used ☐ Not used

Parameters: Multi shell b-values 1000, 2000, and 3000 s/mm2

Preprocessing

Preprocessing software	The HCP minimal processing pipeline (as provided on the connectome database) S1200 release. Followed by processing in MRtrix3 (RC_3) for ODF estimation and tractography.
Normalization	Normalisation was done using the mrtrix population template command for a non-linear group ODF normalization. Tractography was performed both in subject and template space. That template was then further normalised to MNI space along with the resultant streamlines.
Normalization template	Subject specific template + MNI152 for visualisation (provided in supplementary)
Noise and artifact removal	HCP minimal processing pipeline
Volume censoring	HCP minimal processing pipeline

Statistical modeling & inference

Model type and settings	NA
Effect(s) tested	NA

Specify type of analysis: ☐ Whole brain ☐ ROI-based ☐ Both

Statistic type for inference (See Eklund et al. 2016)	NA
Correction	NA

Models & analysis

n/a	Involved in the study
☒	☐ Functional and/or effective connectivity
☒	☐ Graph analysis
☒	☐ Multivariate modeling or predictive analysis
